# Computational Investigation of S781F Mutation-Induced Remodeling of the Staphylococcal Nuclease Domain-Containing Protein 1–Argonaute 2 Interface

**DOI:** 10.3390/ijms27198747

**Published:** 2026-09-30

**Authors:** Jittasak Khowsathit, Yuvaraj Ravikumar, Manu Deeudom, Warinda Prommachote, Bishant Pokharel, Pimpisid Koonyosying, Somdet Srichairatanakool

**Affiliations:** 1 Department of Biochemistry, Faculty of Medicine, Chiang Mai University, Chiang Mai 50200, Thailand; jittasak.khowsathit@cmu.ac.th (J.K.); bishant_p@cmu.ac.th (B.P.); pimpisid.k@cmu.ac.th (P.K.); 2 Department of Biotechnology, School of Sciences and Humanities, SR University, Warangal 506371, India; yuvraj.r@sru.edu.in; 3 Department of Microbiology, Faculty of Medicine, Chiang Mai University, Chiang Mai 50200, Thailand; manu.deeudom@cmu.ac.th; 4 Department of Medical Technology, School of Allied Health Sciences, Walailak University, Nakhon Si Thammarat 80161, Thailand; warinda.pr@wu.ac.th; 5 Center of Excellence Research for Melioidosis and Microorganisms, Walailak University, Nakhon Si Thammarat 80161, Thailand

**Keywords:** SND1, Argonaute 2, S781F mutation, silencing RNA, molecular dynamics simulation, protein–protein interaction, miRNA, cancer

## Abstract

MicroRNA (miRNA)-guided gene silencing depends on the accurate assembly and regulation of the RNA-induced silencing complex (RISC), in which Argonaute-2 (Ago2) interacts with accessory proteins to facilitate complex stability and catalytic activity. Staphylococcal nuclease domain-containing protein 1 (SND1) is an important component of the RISC and a transcriptional co-regulator implicated in multiple cancers; however, the structural consequences of disease-associated SND1 mutations remain poorly understood. In this study, an integrated computational workflow combining protein–protein docking, molecular dynamics simulations, and free energy analyses was employed to investigate the potential structural effects of the SND1 S781F mutation on the SND1–Ago2 interaction. The wild-type complex was predicted to maintain a stable interaction network involving the Tudor domain of SND1 and methylated arginine residues of Ago2. In contrast, the S781F substitution was associated with altered intermolecular hydrogen bonding patterns, reduced predicted interaction stability, increased structural flexibility, altered correlated residue motions, and a broader free energy landscape, suggesting a less stable protein–protein interface. Although the mutant complex sampled a larger number of distinct intermolecular hydrogen bond interactions, these interactions exhibited lower occupancies and reduced persistence than those in the wild-type complex. Collectively, these computational findings suggest that the S781F mutation could act as a potential modulator of the local physicochemical environment, interaction stability, and conformational dynamics of the SND1–Ago2 surface. This study provides atomistic insights into the structural consequences of the S781F mutation and establishes a computational framework for future experimental validation of its role in miRNA-mediated gene regulation and cancer biology.

## 1. Introduction

MicroRNA is involved in post-transcriptional gene regulation and performs a crucial role in governing gene expression, participating in cell proliferation, homeostasis, and tumorigenesis [1]. Essential to this process is the RISC, a multiprotein assembly that mediates miRNA-assisted gene silencing [2]. Ago2, a core component of the RISC, plays a catalytic role in miRNA binding and in guiding the repression of target mRNA [3]. To enable RISC to function efficiently, multiple protein components are necessary to stabilize it [4]. Among them, the SND1 protein, one of the immensely conserved nucleases and transcriptional coactivators, has been recognized for its reader ability [5]. In the RISC, SND1 appears to associate with Ago2 and contribute to gene silencing [6]. Mounting evidence from colocalization and co-immunoprecipitation studies also confirms the physical interaction of SND1 with Ago2, leading to the activation of cancer-relevant pathways [7]. In virtue of its nuclease activity, SND1 increases the efficiency of RISC-guided mRNA degradation, which, in turn, can modulate the expression of specific regulatory transcripts and tumor suppressor genes [8].

In addition to its role in gene silencing, SND1 has been implicated in inflammatory signaling pathways that contribute to cancer progression [9]. In particular, SND1 acts as a transcriptional co-activator of the nuclear factor kappa B (NF-κB) signaling pathway, a pivotal regulator of inflammatory gene expression [10]. In general, NF-κB induction activates the transcription of several genes involved in cell angiogenesis, survival, and metastasis [11]. Earlier studies show that overexpressed SND1 amplifies the NF-κB transcriptional response and activates the expression of oncogene-associated pro-tumorigenic miRNAs and inflammatory mediators [10]. Therefore, uncontrolled SND1 expression has been connected with numerous malignancies, including hepatocellular carcinoma and breast cancer, where it influences tumor invasion and growth and contributes to inflammatory tumor microenvironment development [12]. Furthermore, protein phosphorylation, a key post-translational modification, plays a decisive role in regulating protein stability, function, and intermolecular interactions [13]. Serine residues are the most common targets for phosphorylation, while the phosphorylation of serine is more likely to affect enzymatic function and protein–protein interactions [14]. Importantly, mutations that alter phosphorylatable serine can ultimately alter the modulatory mechanism and may affect RISC assembly [15]. In the case of SND1, serine residues present in the functional “Tudor” domain may serve as regulatory sites that modulate its interaction with its binding partner, as is the case with Ago2 [16].

Of late, cancer genomics studies have identified mutations in SND1, including the substitution of serine at position 781 with phenylalanine (S781F) [17]. Because phenylalanine lacks the hydroxyl group necessary for phosphorylation, this mutation might abrogate phosphorylation [18]. The absence of phosphorylation at serine residues has been commonly reported to be able to remove a residue that could primarily serve as a phosphorylation site with protein interactions and inflammatory signaling events involved in tumor control [19]. Modifications to phosphorylatable residues can disrupt normal cellular signaling [20]. For example, phosphorylation-dependent modulation of tumor suppressor proteins, including phosphatase and tensin homolog (PTEN), plays a crucial role in regulating cellular homeostasis [21]. The abolishment of PTEN phosphorylation has been linked to the activation of the phosphatidylinositol 3-kinase (PI3K)/protein kinase B (AKT) pathway, which, in turn, activates NF-κB signaling and inflammatory responses, thereby favoring the tumor microenvironment [21,22]. Likewise, loss or dysregulation of forkhead box O3a (FOXO3a) phosphorylation can perturb its transcriptional activity, subsequently leading to increased NF-κB-mediated gene expression, which is responsible for tumor progression and inflammation [23]. Owing to these reported findings, the S781F mutation might also alter the structural and functional properties of SND1, potentially by disrupting its interaction with Ago2 and the assembly of the RISC [6]. In addition, higher levels of free SND1 could induce NF-κB signaling, thereby enhancing the expression of downstream targets, including pro-angiogenic factors and inflammatory cytokines [24]. As is well known, continuous activation of NF-κB signaling is commonly observed in tumor-triggered inflammation, the promotion of metastasis, and increased cell survival [25]. Thus, SND1 S781F may alter its functional role from RISC-controlled RNA silencing to the transcriptional stimulation of inflammatory pathways [26]. In light of this and given its high potential, it is worth investigating the structural and functional consequences of the SND1 S781F mutation [27]. As experimental evidence supporting this mutation-mediated structural and interaction changes is challenging and time-consuming to obtain, we sought to use computational approaches to elucidate this mechanistic hypothesis [28].

In recent times, in silico methods, such as molecular docking and molecular simulations, have become valuable tools for examining protein structural stability, protein–protein interactions, and conformational dynamics on an atomistic scale [29]. These methods aid researchers in probing how specific mutations influence protein-binding affinity for interacting partners and protein folding [30]. In accordance with SND1, the S781F mutation may cause local structural perturbations in the Tudor domain, possibly disrupting its interaction with Ago2 and altering RISC stability [31]. Molecular docking approaches can yield insights into the SND1–Ago2 binding interface and interactions, while molecular dynamics (MD) simulation can subsequently explain the time-dependent stability and conformational changes between wild-type and mutant SND1 [32]. Although this study investigated S781F substitution, a residue lacking a phosphorylatable hydroxyl group, the computational analyses compared unphosphorylated Ser781 with Phe781 and therefore did not directly assess the consequences of phosphorylation loss. Nevertheless, the site-specific phosphorylation of SND1 at Ser781 and its potential influence on Ago2 recognition were not experimentally performed in this study. Consequently, this current work is limited to the structural and physicochemical consequences of Ser-to-Phe substitution without drawing any conclusions related to phosphorylation-related regulation [33].

## 2. Results

### 2.1. Molecular Docking

**Figure 1:**
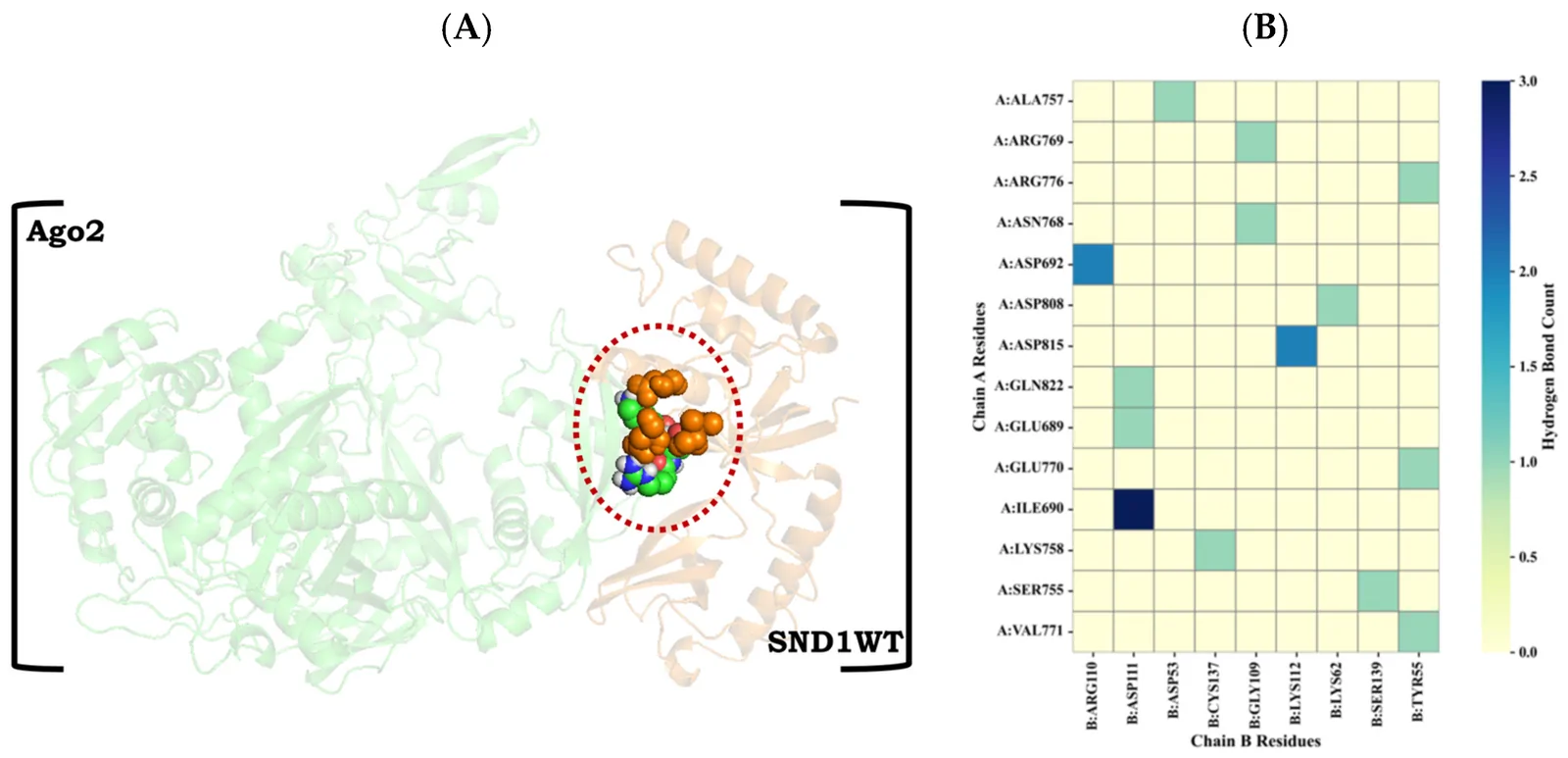

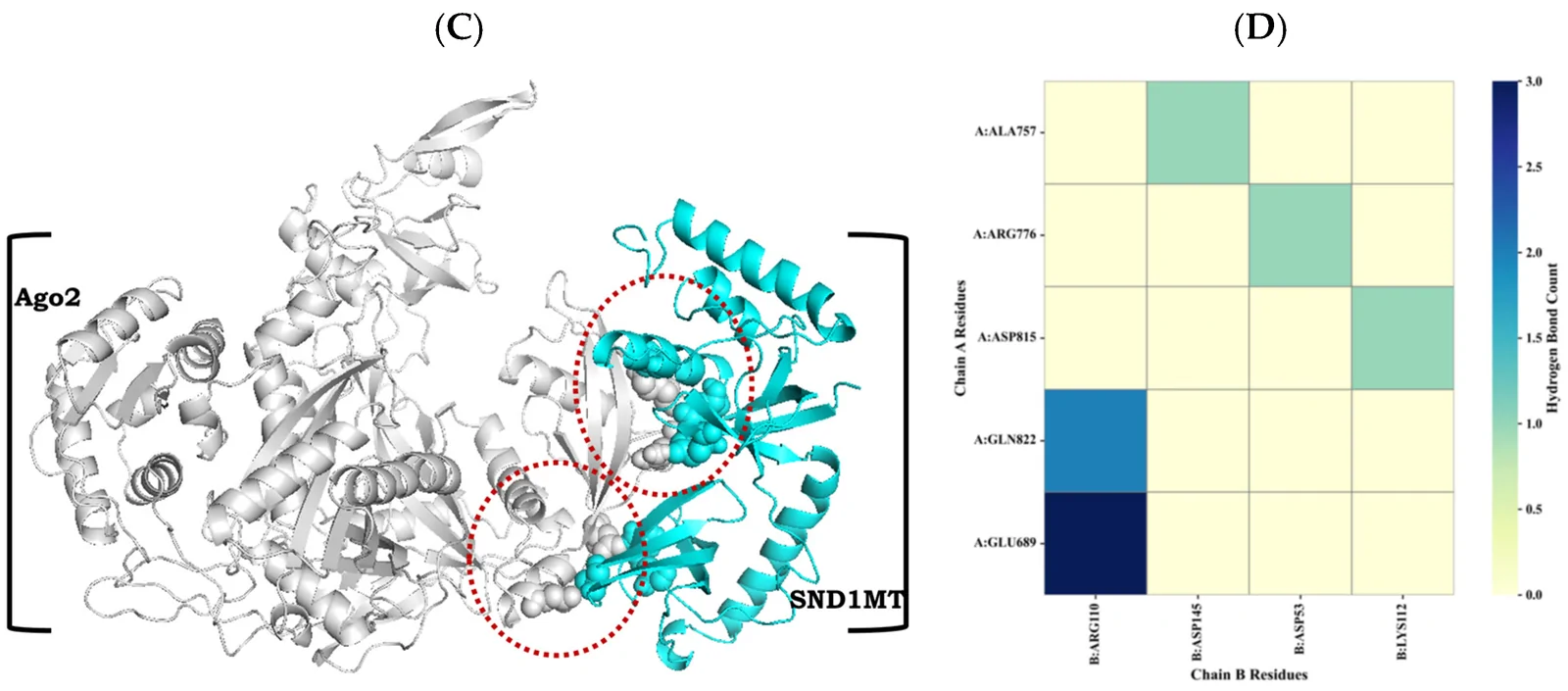
Structural overview and molecular docking analysis of the wild-type staphylococcal nuclease domain-containing protein 1 (SND1WT)–Argonaute 2 (Ago2) and S781F mutant staphylococcal nuclease domain-containing protein 1 (SND1MT)–Ago2 complexes. The docking models shown are representative structures selected from the highest-ranking High Ambiguity Driven Biomolecular DOCKing (HADDOCK) clusters. (**A**) Three-dimensional representation of the docked SND1WT–Ago2 complex generated using HADDOCK, with Ago2 shown in green and SND1WT in salmon/orange, with residues participating in protein–protein interface highlighted in red circle. (**B**) Intermolecular hydrogen bond map for the SND1WT–Ago2 complex, showing hydrogen bond counts (color scale, 0–3) between each SND1 residue (chain A, rows; 14 residues) and Ago2 residue (chain B, columns; 9 residues) in the representative docked pose. (**C**) Three-dimensional representation of the docked SND1MT–Ago2 complex, with Ago2 shown in gray and SND1MT in cyan, with resides participating in protein–protein interface highlighted in red circles. (**D**) Intermolecular hydrogen bond map for the SND1MT–Ago2 complex.

The molecular docking of SND1-wild type (SND1WT) and the S781F mutant (MT) variation (SND1MT) was performed against Ago2 using the Haddock software 2.4. The docking results for the protein–protein interaction are depicted in Figure 1. The High Ambiguity Driven Biomolecular DOCKing (HADDOCK) score was determined based on the energy attained from the sum of desolvation energy, electrostatic intermolecular energy, and van der Waals energy. In addition, the Z-score, which represents the number of standard deviations, was calculated. The van der Waals and electrostatic energy values were recorded at −43.2 ± 6.7 and −255.3 ± 48.9 for SND1WT, and −32.6 ± 4.8 and −277.4 ± 18.1 for SND1MT, respectively. Further, to assess the proportion of the protein surface not in contact with water, the Buried Surface Area (BSA) parameter was considered. A higher BSA value of 2.4 ± 1.1 was observed in SND1MT, while SND1WT showed a lower BSA value of 1.2 ± 1.7. The SND1WT–Ago2 complex showed a docking score of −86.6 ± 9.0 arbitrary units after having interactions with certain residues such as Ala757, Arg769, Arg776, Asn768, Asp692, Asp808, Asp815, Gln822, Glu689, Glu770, Ile690, Lys758, Ser755, and Val771. The SND1S781F-Ago2 complex exhibited a marginally less favorable docking score of −85.5 ± 5.3 arbitrary units after having crucial interactions with Ala757, Arg776, Asp815, Gln822, and Glu689. It is also noteworthy to mention that the total number of interactions was higher in the WT (SND1–14 and Ago2–9) than in the MT (SND1–5 and Ago2–4). Overall, HADDOCK resulted in numerous clusters for both SND1WT–Ago2 and SND1MT–Ago2. The HADDOCK score is generally considered a composite score, and the values attained here cannot be taken as an exact binding free energy score. The mean score between the SND1WT–Ago2 and SND1MT–Ago2 appears to be minimal, while the cluster scores seem to overlap significantly. Quantitatively, the 1.1-unit gap between the two mean scores was small relative to the corresponding standard deviations (9.0 and 5.3), and we therefore did not interpret it as a meaningful energetic distinction between the wild-type and mutant complexes. Notably, the more informative structural observation would be the difference in the number of predicted interfacial contacts noted above. We also note explicitly that Ser781 itself did not appear among the predicted interfacial contact residues listed above, nor among the aromatic-cage residues (Phe740, Tyr746, Tyr763, and Tyr766) that are capable of recognizing dimethylated arginine (DMA). Importantly, the structural basis by which the S781F substitution can nevertheless perturb this interface will be addressed in the Discussion. Further, the interactions displayed between these two proteins at the interface were compared with the existing structural interaction of SND1 with the DMA (3OMC). It was found that the experimentally demonstrated SND1 Tudor domain interacts with the dimethyl arginine through an aromatic cage and residues lying in the SN-domain. The obtained results were therefore plausibly considered to be correct, as the interactions of SND1, both WT and MT, were in a similar orientation and recognition groove, recognizing the DMA of Ago2. Thus, without considering only the docking score, the corresponding models that showed the highest-ranking clusters and had appropriate interactions from the SND1 active site were subsequently used as a starting structure for MD simulation studies.

### 2.2. Molecular Dynamics Simulation

All-atom MD simulation was performed to assess the effect of the S781F mutation on the SND1 protein and its complexes, focusing on their conformational changes and stability over a 200 ns period. This study comprised six systems: (1) isolated SND1WT; (2) isolated SND1MT; (3) isolated, unmodified Ago2 (Ago2); (4) isolated Ago2 carrying the DMA at residue 305 (Ago2–DMA); (5) the SND1WT–Ago2–DMA complex (SND1WT–Ago2); and (6) the SND1MT–Ago2–DMA complex (SND1MT–Ago2). It is also important to note that each system was simulated using a single 200 ns trajectory. Hence, the results interpreted below serve only as exploratory and hypothesis-generating results rather than concrete evidence of altered equilibrium affinity between the various systems.

### 2.3. Root Mean Square Deviation Analysis

**Figure 2:**
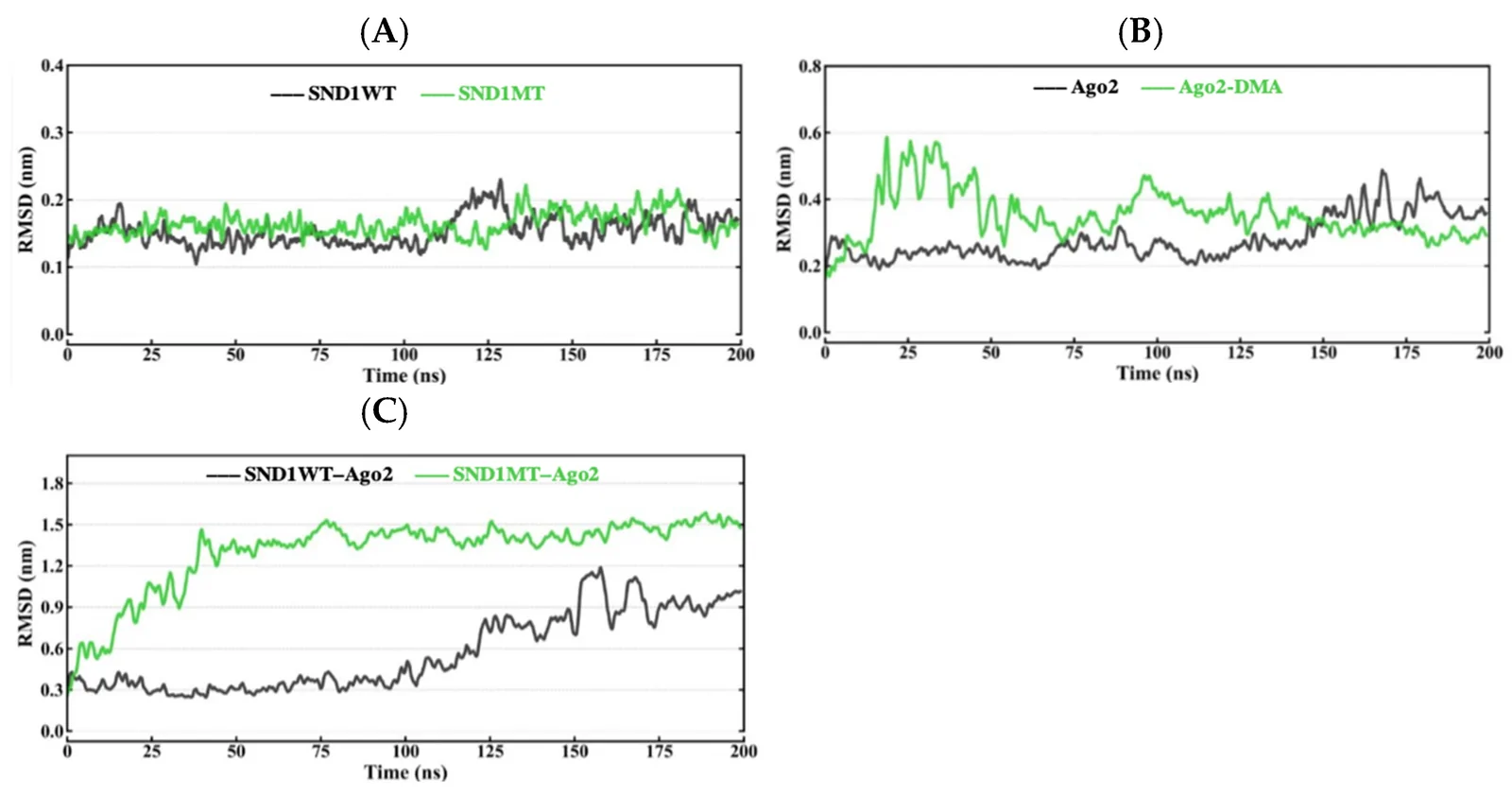
Root mean square deviation (RMSD) analysis of SND1, Ago2, and Ago2-bound complexes during 200 ns molecular dynamics (MD) simulations. (**A**) Backbone RMSD trajectories of SND1WT (black) and SND1MT (green). (**B**) RMSD profiles of unmodified Ago2 (black) and dimethylated arginine-containing Ago2 (Ago2–DMA; green) systems. (**C**) Backbone RMSD trajectories of the SND1WT–Ago2 (black) and SND1MT–Ago2 (green) complexes.

The root mean square deviation (RMSD) plot, which reveals structural differences among the different protein forms over a 200 ns simulation, provided crucial insight into the stability of both the wild-type and mutant forms of SND1 in their free and Ago2-bound states. In SND1WT and SND1MT, no substantial fluctuations were observed. The average RMSD values for both forms were very small (0.15 ± 0.03 nm and 0.16 ± 0.02 nm), differing by less than 0.1 nm. Similar RMSD values were measured for Ago2 (0.28 ± 0.07 nm) and Ago2–DMA (0.35 ± 0.08 nm). As depicted in Figure 2, modest RMSD fluctuations were observed, particularly for Ago2–DMA (~0.2 nm). The system showed greater fluctuations during the initial 50 ns. For the Ago2-bound forms, the average RMSD values of the SND1WT–Ago2 and SND1MT–Ago2 complexes were 0.56 ± 0.28 nm and 1.30 ± 0.27 nm, respectively. These values indicate the overall stability of all systems: Ago2-free WT and MT did not differ significantly from their initial conformations, whereas Ago2-bound WT and MT exhibited considerable fluctuations. In the initial time frame, the RMSD fluctuations for Ago2 were ~0.2 nm, while they were ~0.6 nm for Ago2–DMA. From these observations, the S781F substitution was associated with larger backbone deviations in the SND1MT–Ago2 complex than in the wild-type complex. We note, however, that neither complex trajectory reached an unambiguous plateau within the 200 ns window, as the SND1WT–Ago2 trace in particular continued to drift over the final third of the simulation. Consequently, these RMSD values should be read as evidence of ongoing interfacial rearrangement rather than as converged equilibrium properties.

### 2.4. Root Mean Square Fluctuation Analysis

**Figure 3:**
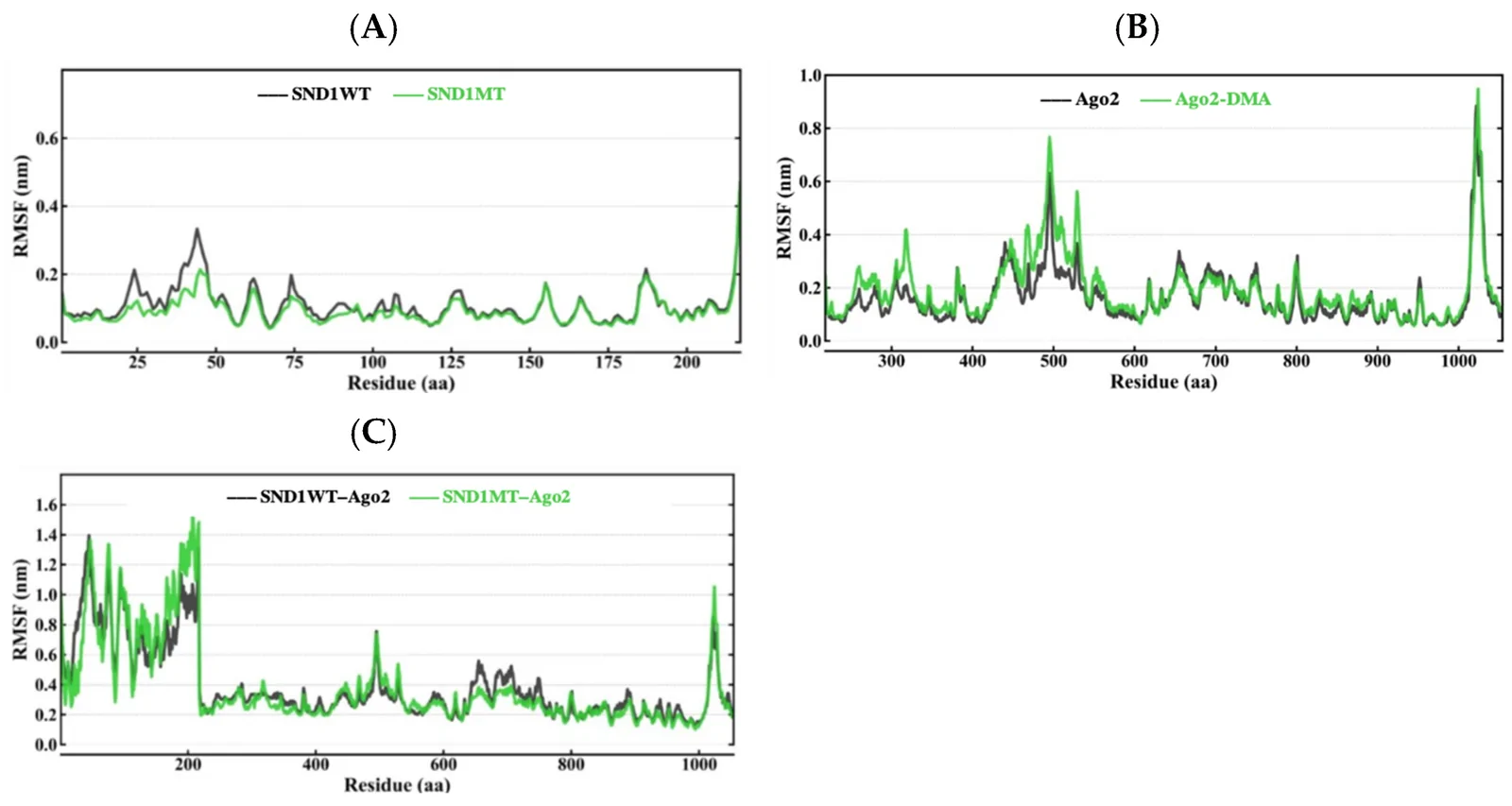
Root mean square fluctuation (RMSF) analysis of residue-level flexibility changes in the wild-type (WT) and S781F mutant (MT) systems during the 200 ns molecular dynamics (MD) simulations. (**A**) RMSF profiles of SND1WT (black) and SND1MT (green). (**B**) Residue fluctuation patterns of Ago2 (black) and Ago2–DMA (green). (**C**) RMSF profiles of SND1WT–Ago2 (black) and SND1MT–Ago2 (green) complexes. RMSF values were calculated for the protein backbone atoms over the simulation trajectory.

Root mean square fluctuation (RMSF) analysis reports the deviation of each residue from its time-averaged position and is widely used to describe residue-level flexibility. The RMSF profiles for the isolated proteins and for the SND1–Ago2 complexes are compared in Figure 3. Among the isolated systems, average RMSF values were uniformly low: SND1WT (0.11 ± 0.05 nm), SND1MT (0.09 ± 0.04 nm), Ago2 (0.16 ± 0.10 nm), and Ago2–DMA (0.19 ± 0.12 nm). Average RMSF values were substantially higher, but very similar to one another, for the two complexes: 0.39 ± 0.24 nm for SND1WT–Ago2 and 0.39 ± 0.29 nm for SND1MT–Ago2. The mutant complex additionally showed marginally larger fluctuations than the wild-type complex in several loop regions. It should also be noted that RMSF values for the complexes were computed after fitting the entire two-protein assembly, whereas those for the isolated proteins were fitted individually. Consequently, part of the apparent increase on binding reflected relative inter-protein (hinge-like) motion rather than a uniform increase in local disorder, whereas absolute values were therefore not directly comparable between the two groups. Within the complexes, where the fitting procedure was identical, the modestly higher fluctuations and more localized high-flexibility loops of SND1MT–Ago2 were consistent with the increased conformational variability indicated by the RMSD, hydrogen bond, and free energy landscape analyses described below.

### 2.5. Radius of Gyration and Solvent Accessible Surface Area

**Figure 4:**
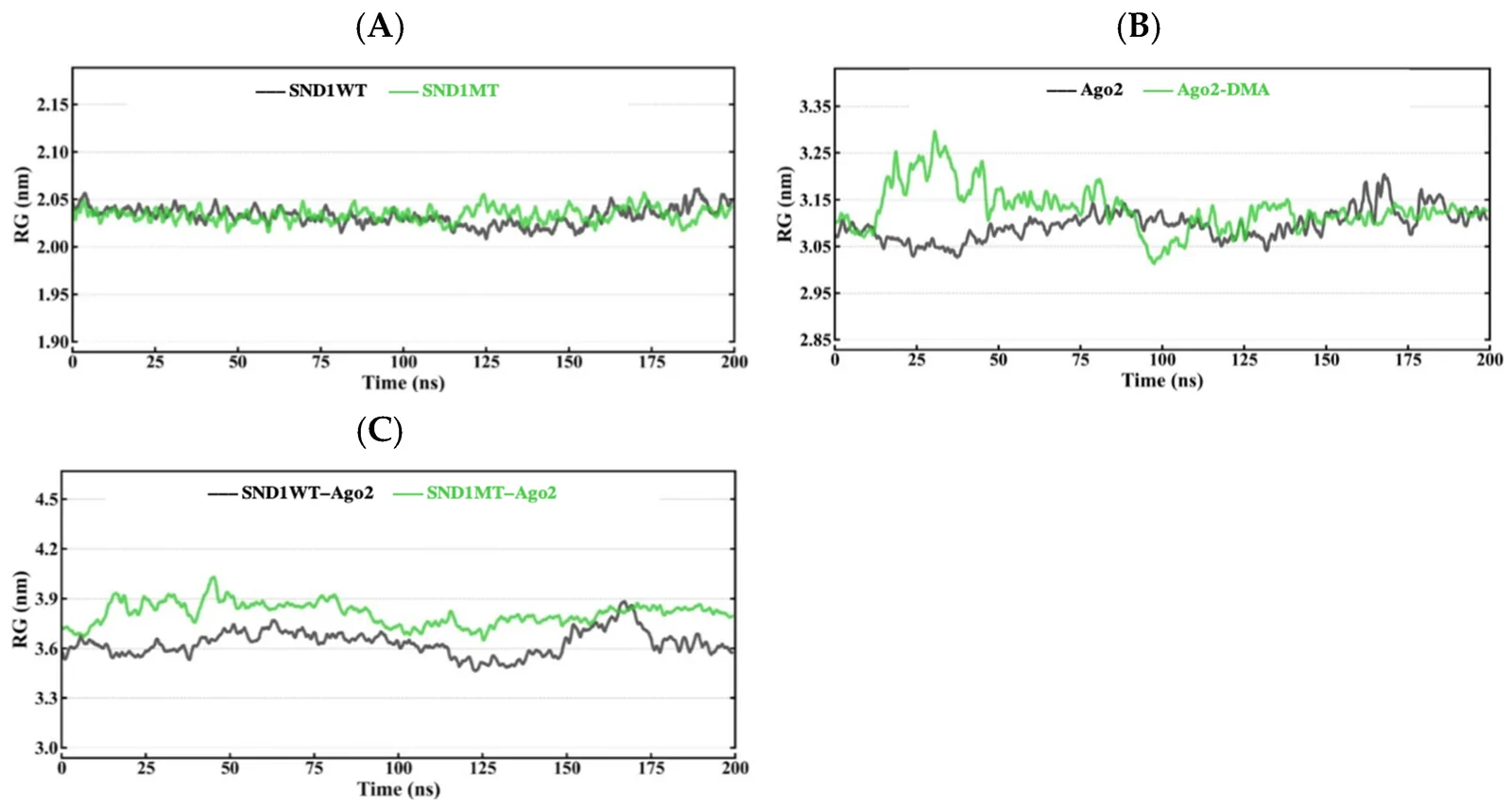
Radius of gyration (Rg) analysis during the 200 ns MD simulations. (**A**) Radius of gyration trajectories of isolated SND1WT (black) and SND1MT (green). (**B**) Rg profiles of unmodified Ago2 (black) and Ago2–DMA (green). (**C**) Rg analysis of SND1WT–Ago2 (black) and SND1MT (green)–Ago2 complexes. Rg values were calculated from the protein backbone atoms throughout the simulation.

Radius of gyration (Rg) is a vital parameter that quantifies the distribution of a protein’s mass around its center of mass, reflecting its overall compactness. A higher Rg value indicates a more dispersed conformation, while a lower Rg value suggests a more compact structure. In this study (Figure 4), for both SND1WT and SND1MT, the Rg values were 2.03 ± 0.01 nm, indicating a more compact structure. For Ago2 (3.10 ± 0.03 nm) and Ago2–DMA (3.13 ± 0.05 nm), the mean Rg values showed mild fluctuations. Notably, during the initial 75 ns, Rg values increased by ~0.22 nm between Ago2 and Ago2–DMA, after which they reached a steady equilibrium. When compared with the unbound state of SND1 and Ago2, the largest Rg values in this study were recorded for the two complexes, SND1WT–Ago2 (3.63 ± 0.08 nm) and SND1MT–Ago2 (3.81 ± 0.07 nm), as would be expected for a larger two-protein assembly. Minimal changes in Rg in the unbound Ago2 (both WT and MT) suggest that methylated arginine does not affect the overall structural compactness of the protein. Likewise, the differences between Ago2-bound SND1WT and SND1MT (~0.18 nm) did not appear substantial enough to indicate significant changes in protein compactness, even after protein–protein interaction. In quantitative terms, this ~0.18 nm difference between the two complexes corresponds to roughly 5% of their mean Rg and is therefore treated here as a small trend that was consistent with, but not independently diagnostic of, the more expanded mutant ensemble suggested by the other descriptors.

**Figure 5:**
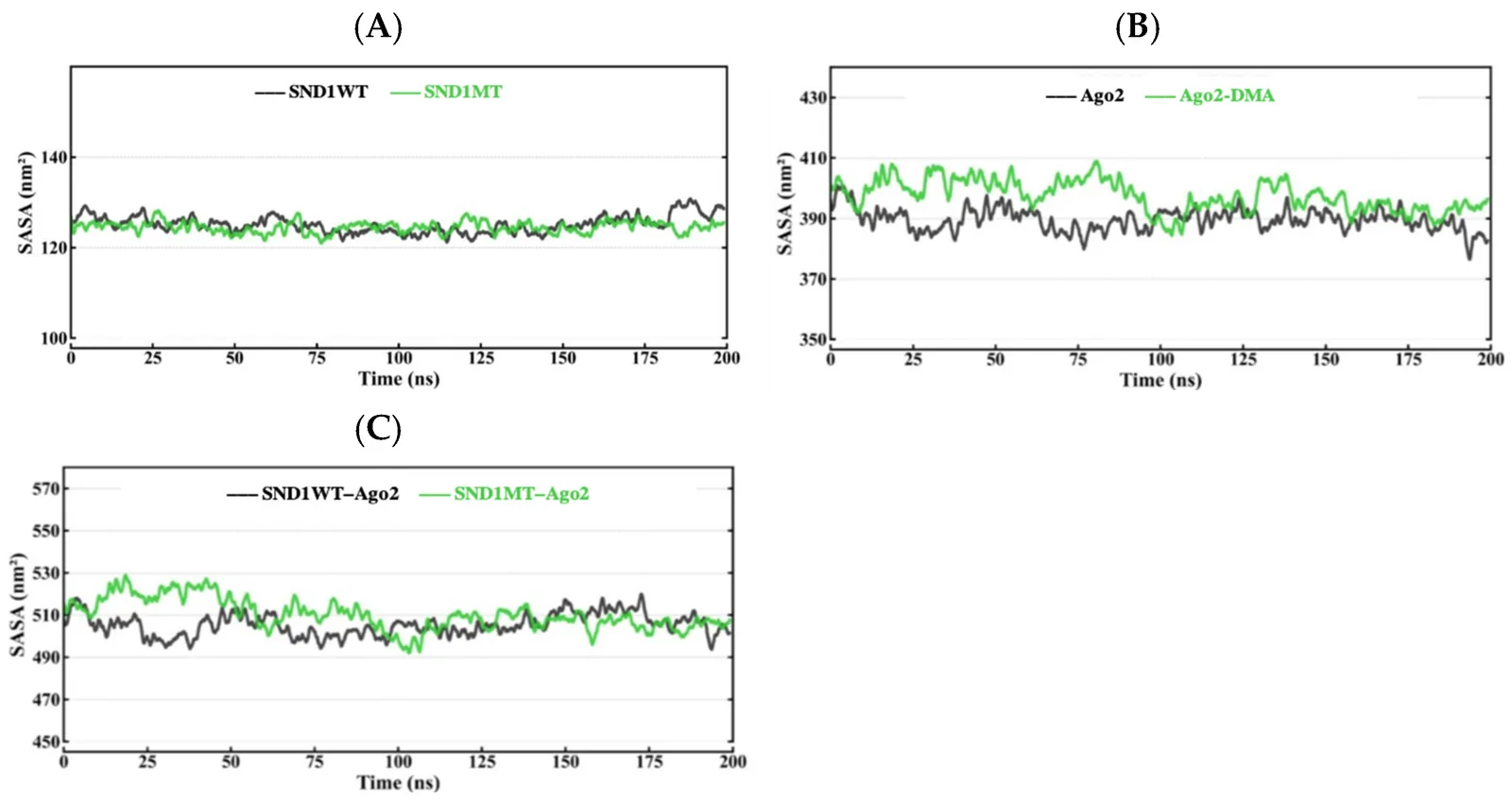
Solvent-accessible surface area (SASA) analysis during the 200 ns MD simulations. (**A**) SASA trajectories of isolated SND1WT (black) and SND1MT (green). (**B**) SASA profiles of Ago2 (black) and Ago2–DMA (green). (**C**) SASA analysis of SND1WT–Ago2 (black) and SND1MT-Ago2 (green) complexes. SASA values were calculated over the 200 ns molecular dynamics simulations.

On the other hand, solvent accessible surface area (SASA) indicates the extent to which the protein surface is exposed to the surrounding solvent. Differences in SASA values suggest structural destabilization, thereby weakening protein interactions with other proteins or ligands (Figure 5). For SND1WT (125.16 ± 2.37 nm^2^) and SND1MT (124.51 ± 1.90 nm^2^), the measured SASA values were almost identical at ~125 nm^2^, indicating a less-exposed structure, whereas in Ago2 (389.52 ± 4.74 nm^2^) and Ago2–DMA (397.51 ± 5.91 nm^2^), the SASA values increased slightly. In Ago2, the values were ~380–390 nm^2^, and in Ago2–DMA, the increase was slightly greater, with values in the range of 390–410 nm^2^. This result indicates that Ago2, both wild-type and arginine-methylated forms, was moderately exposed to the solvent. Finally, the SND1WT–Ago2 and SND1MT–Ago2, complexes showed SASA values of 505.32 ± 6.23 nm^2^ and 510.23 ± 8.02 nm^2^, respectively. This result suggests slightly greater solvent exposure when the mutant SND1 interacted with Ago2.

### 2.6. Inter-Hydrogen Bond Analysis

**Figure 6:**
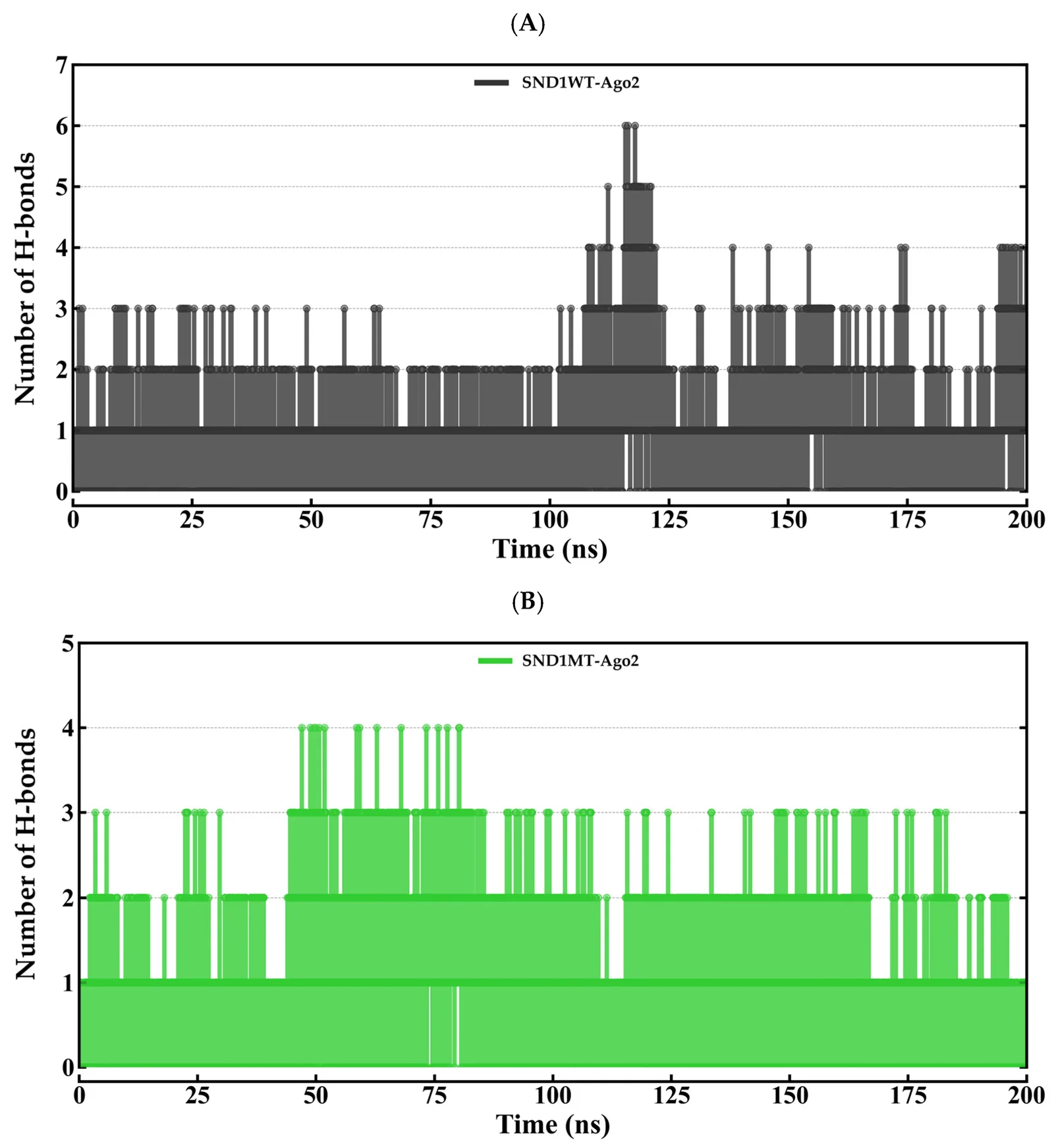
Intermolecular hydrogen bond analysis during the 200 ns MD simulations. (**A**) Time evolution of the number of intermolecular hydrogen bonds between SND1 and Ago2 in the SND1WT–Ago2 complex (black). (**B**) Time evolution of the number of intermolecular hydrogen bonds between SND1 and Ago2 in the SND1MT–Ago2 complex (green). Hydrogen bonds were monitored throughout the 200 ns MD simulation.

The time evolution values of the number of hydrogen bonds between SND1WT–Ago2 and SND1MT–Ago2 for 200 ns are shown in Figure 6. In general, hydrogen bonding is a decisive factor in preserving the stability of protein–protein interactions. In the SND1WT–Ago2 system, the number of hydrogen bonds oscillates between three and four, occasionally reaching six, indicating a strong, stable hydrogen bonding network. In contrast, SND1MT–Ago2 displayed a minimal number of hydrogen bonds, generally one to three, occasionally four, suggesting that the S781F mutation may diminish specific regions of the protein, thereby compromising its structural integrity. This finding remains crucial to understanding the stability between the two interacting proteins. Furthermore, intramolecular H-bond analysis was conducted to evaluate the impact of the mutation on protein–protein interaction and on SND1 alone.

**Figure 7:**
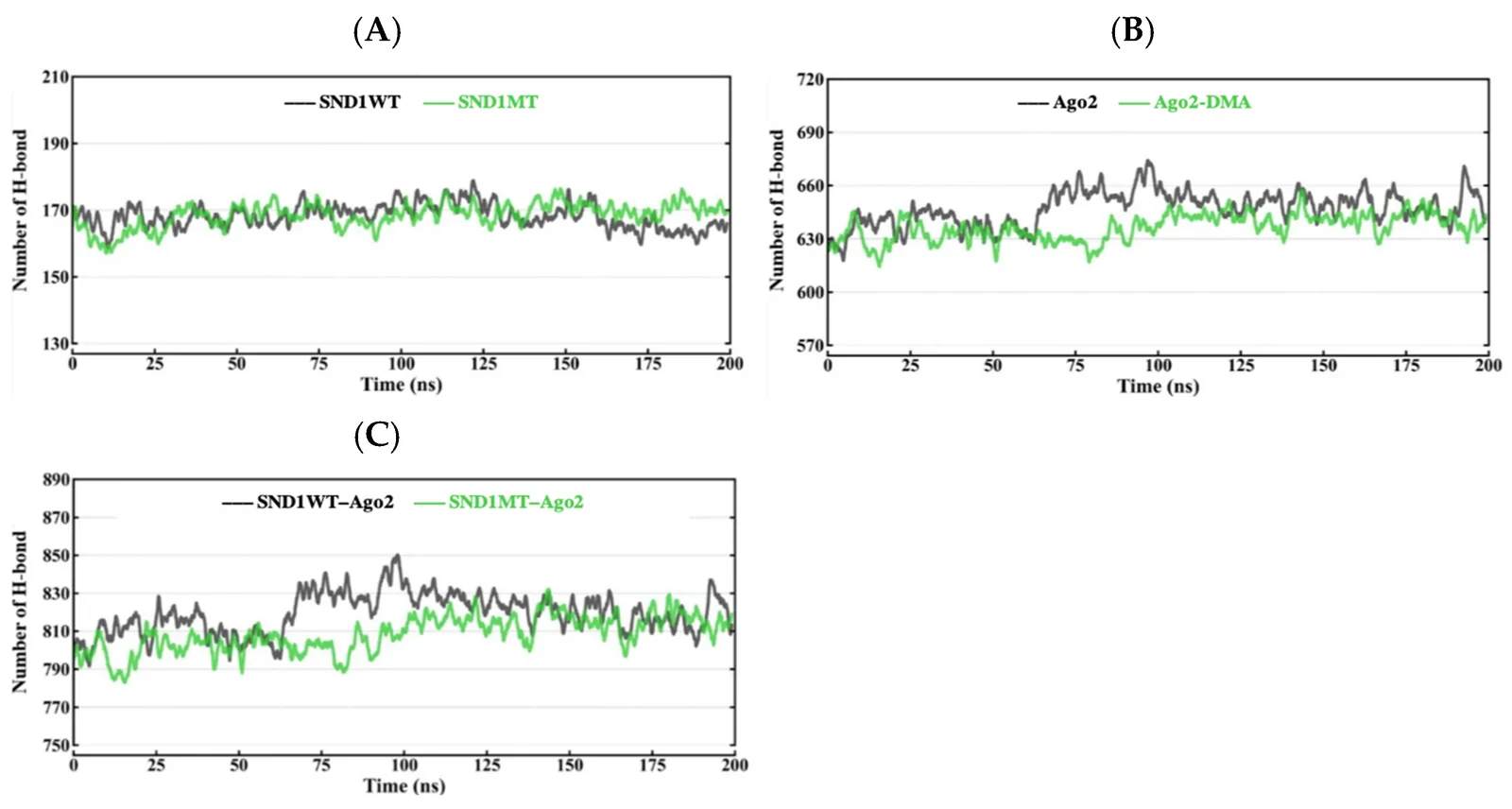
Intramolecular hydrogen bond analysis during the 200 ns MD simulations. (**A**) Time evolution of the number of intramolecular hydrogen bonds in SND1WT (black) and SND1MT (green). (**B**) Time evolution of the number of intramolecular hydrogen bonds in Ago2 (black) and Ago2–DMA (green). (**C**) Time evolution of the number of intramolecular hydrogen bonds in the SND1WT–Ago2 complex (black) and the SND1MT-Ago2 complex (green). Hydrogen bonds were monitored throughout the 200 ns MD simulation.

As shown in Figure 7, the S781F substitution in SND1 was associated with a reduction in intramolecular H-bonds within the protein. On average, SND1MT maintained approximately 11 fewer intramolecular H-bonds than SND1WT over the 200 ns simulation (mean H-bond count ~170 for SND1WT versus ~165 for SND1MT). A similar trend was observed for the complexes, where the mean H-bond count was lower for SND1MT–Ago2 (~808) than for SND1WT–Ago2 (~818).

### 2.7. Principal Component Analysis

**Figure 8:**
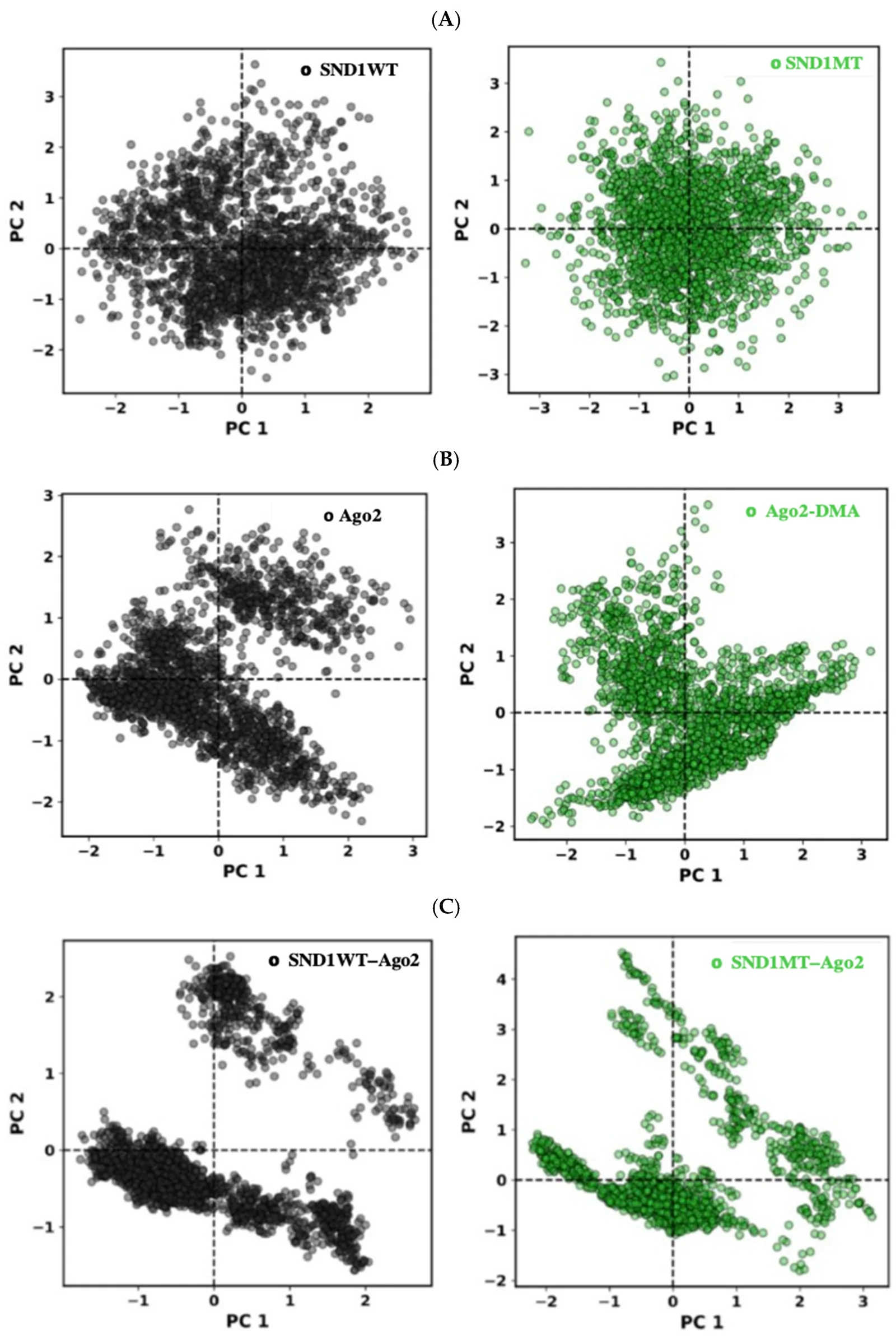
Principal component (PC) analysis of conformational motions during the 200 ns MD simulations. (**A**) Projection of SND1WT (black) and SND1MT (green). (**B**) Projection of unmodified Ago2 (black) and Ago2–DMA (green). (**C**) Projection of the SND1WT–Ago2 (black) and SND1MT-Ago2 (green) trajectories onto PC1 and PC2. Principal components were calculated from the protein backbone atomic coordinates obtained during a 200 ns MD simulation.

To probe the collective motions in SND1WT, SND1MT, Ago2, Ago2–DMA, SND1WT–Ago2, and SND1MT–Ago2, we carried out principal component (PC) analysis using the few eigenvectors that tend to explain the global motions of a protein. The conformational dynamics of the above-mentioned proteins are presented in Figure 8. As depicted in Figure 8, most of the variance is regularly dispersed along principal components 1 (PC1) and 2 (PC2). The lack of distinct clusters suggests that conformations are likely to be in a single state with limited structural deviations. A similar pattern is observed for SND1MT, indicating that the S781F mutation (SND1MT) did not significantly alter the structure. In Ago2 and Ago2–DMA, the variances are more organized into multiple forms and appear to move away from the center. In particular, the clusters are even more widely distributed in Ago2–DMA than in Ago2, where they are comparatively compact. These observations suggest that arginine methylation induces perturbation, enabling the protein to undergo multiple transitional states. Compared with the unbound state, clearly scattered, dominant clusters are seen along the axis. In the SND1WT–Ago2, a few sets were found, although isolated, yet remained compact, indicating the existence of defined conformational states. In the SND1MT–Ago2, the variance sets are highly expanded, uneven, and diffused throughout. Such widely dispersed, broad clusters over 200 ns were much lower given the limited sampling and absence of full convergence; however, these regions cannot be interpreted as well-defined thermodynamic metastable states. Taken together, the PC analysis indicates that S781F mutations are associated with greater conformational heterogeneity, thereby delineating the overall stability of protein–protein interaction.

### 2.8. Free Energy Landscape Analysis

**Figure 9:**
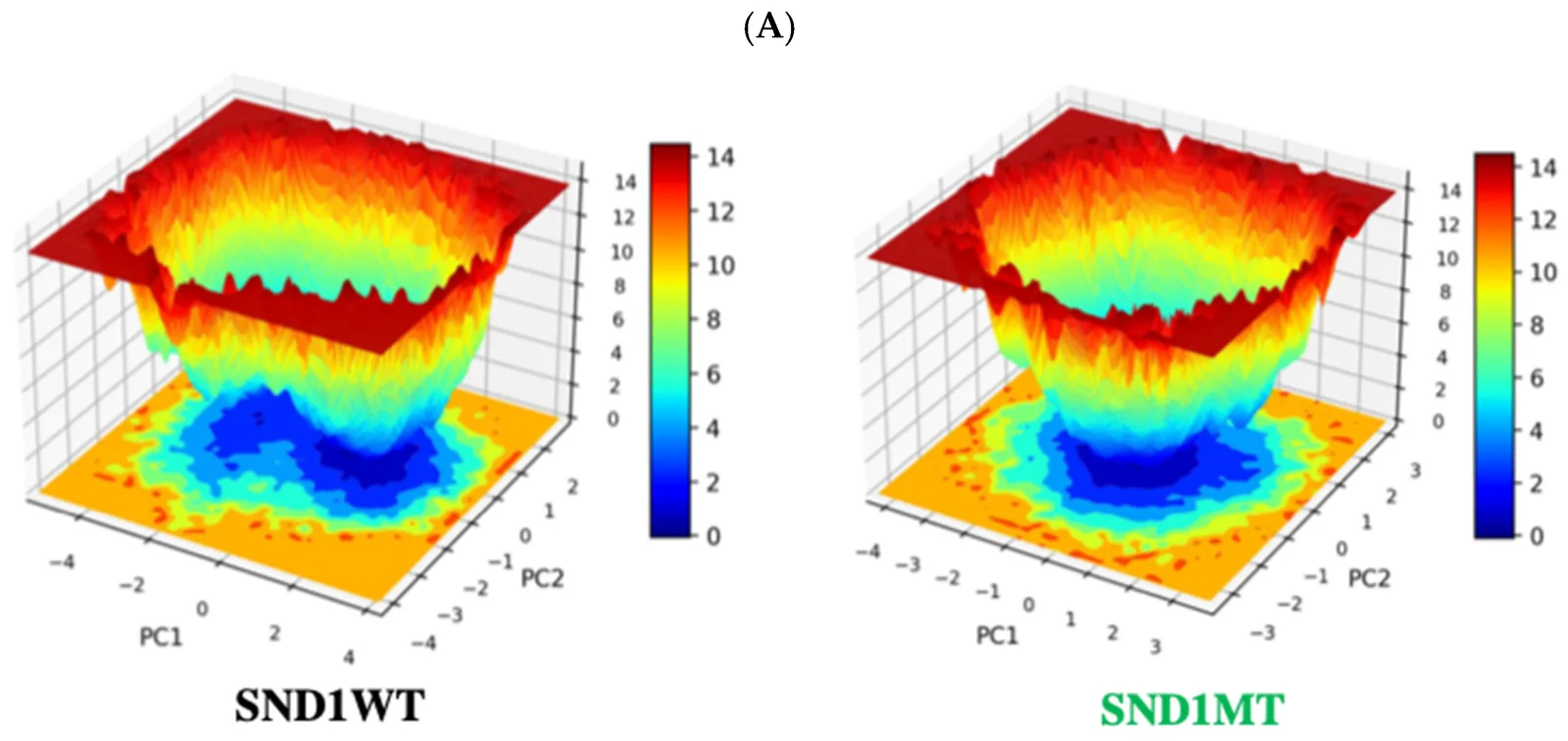

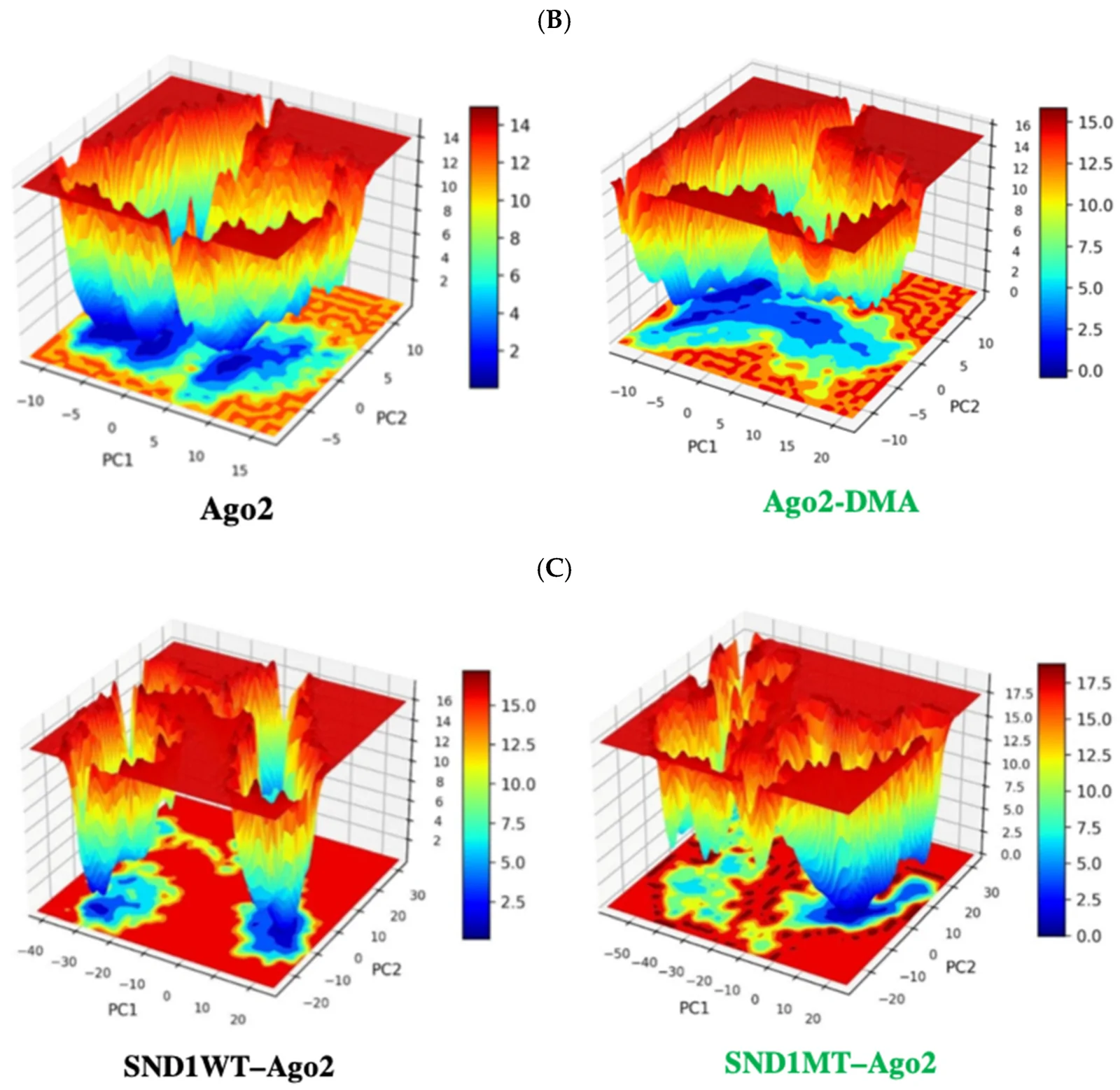
Free energy landscape (FEL) analysis of the WT and S781F mutant systems during the 200 ns MD simulations. (**A**) FEL plots of SND1WT and SND1MT. (**B**) FEL plots of Ago2 and Ago2–DMA. (**C**) FEL plots of SND1WT–Ago2 and SND1MT-Ago2 complexes projected along PC1 and PC2 coordinates. The FEL plots were constructed using the first two principal components (PC1 and PC2) as reaction coordinates. The color scale represents the relative free energy, with blue indicating low-energy conformational states and red indicating high-energy conformational states.

The free energy landscape (FEL) analysis for the SND1WT, SND1MT, Ago2, Ago2–DMA, SND1WT–Ago2, and SND1MT–Ago2 was plotted using their respective PC1 and PC2 values. From Figure 9, the color palette indicates free energy: unstable states (high energy) are shown in red, intermediate states in green to yellow, and stable conformational states in blue. In SND1WT, a predominant energy basin in the form of a funnel was observed. This appearance implies that a favorable conformational state prevails for most of the simulation time. In SND1MT, a mildly wider and slightly irregular landscape is observed, indicating a moderate broadening of the conformational space, although the global energy tended to remain largely the same. In Ago2 and Ago2–DMA, numerous low-energy basins are observed mixed with higher-energy basins. In Ago2, at least two to three distinct funnels are observed, indicating that the protein adopts different conformational states over the course of the simulation. With respect to the Ago2–DMA, an unorganized, wider basin with few blunt funnels was most frequently observed. This pattern indicates that arginine methylation of Ago2 increases its conformational flexibility, resulting in multiple structural states. Finally, the maximum substantial divergence in conformational states and energy distribution was noted in the Ago2-bound systems. This is consistent with increased conformational variability but does not by itself establish differences in thermodynamic stability. The presence of a few distinct basins indicates that the transitions are limited and that the conformational states are better organized and more structured. In contrast, in the SND1MT–Ago2 systems, highly irregular, separated, and broader basins were found. The low-energy regions were more scattered across the PC1 and PC2 coordinates. Because the overall energy scale was comparable between the wild-type and mutant landscapes, this difference is described here as a redistribution and broadening of the sampled conformational space. Consequently, the change in which conformations were visited, and how distinct they were from one another, should be acknowledged. However, they should not be seen as evidence of a change in the underlying energetics of the interface. Overall, the FEL shows a more focused low-energy region for WT and a broader, more fragmented low-energy distribution for SND1MT-Ago2. Also, as the trajectories are not fully converged, the FEL plots can be interpreted in terms of qualitative rather than quantitative thermodynamic free energy landscapes.

### 2.9. Dynamic Cross-Correlation Matrix Analysis

**Figure 10:**
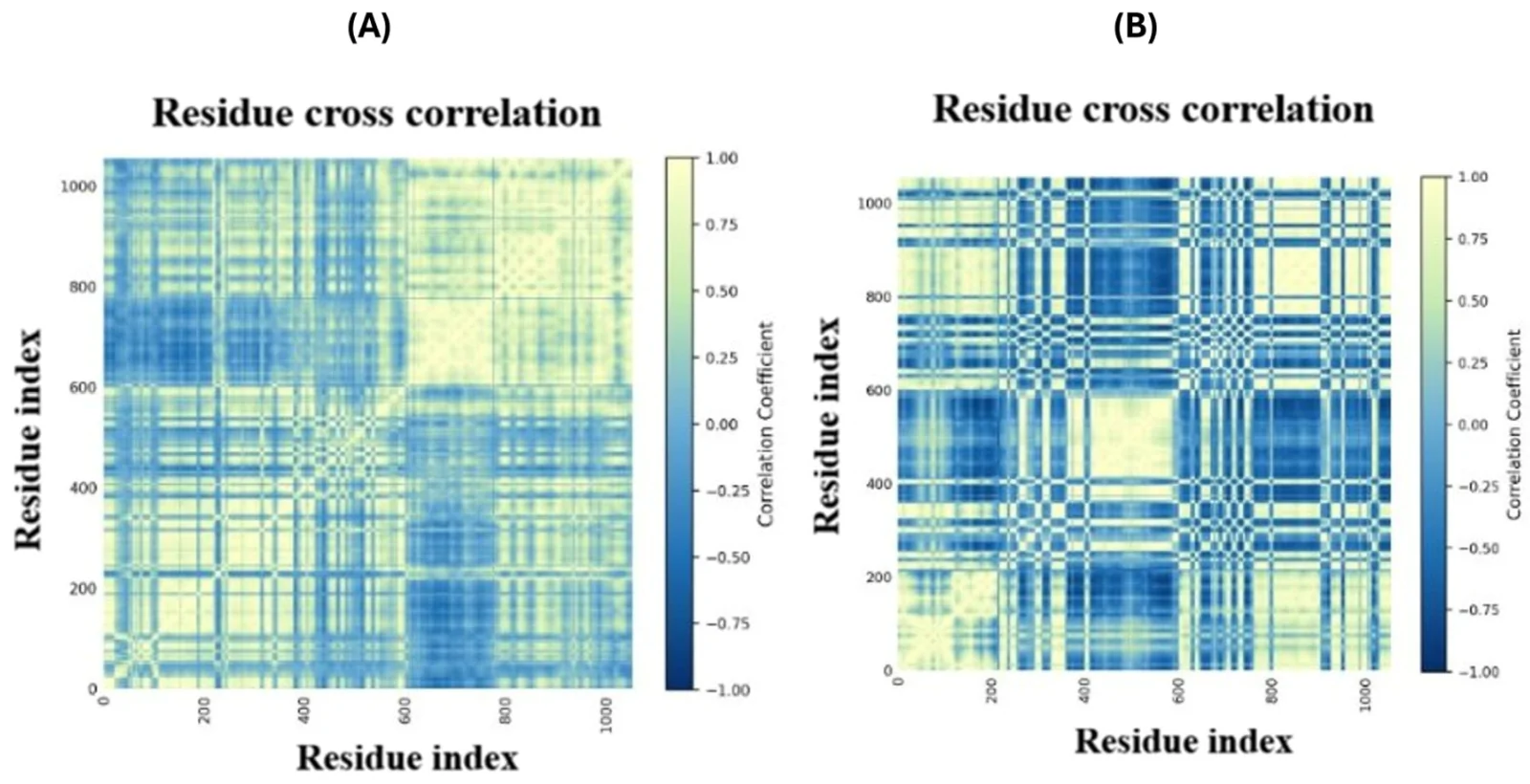
Dynamic cross-correlation matrix (DCCM) analysis of residue motions during the 200 ns MD simulations. DCCM was calculated from the Cα-atom fluctuations obtained from the MD trajectories of (**A**) the SND1WT–Ago2 complex and (**B**) the SND1MT–Ago2 complex. The color scale represents the cross-correlation coefficient, where positive values (yellow) indicate correlated motions, negative values (blue) indicate anticorrelated motions, and values near zero indicate weak or no correlation.

Dynamic cross-correlation matrix (DCCM) analysis was performed to evaluate the correlated motions of residues during the 200 ns molecular dynamics simulations and to compare the dynamic coupling between the wild-type and mutant SND1–Ago2 complexes. Positive correlation values represent residues moving in the same direction, whereas negative correlation values indicate residues exhibiting opposite or anticorrelated motions. As shown in Figure 10A, the SND1WT–Ago2 complex exhibited extensive regions of positive correlation (yellow), indicating coordinated residue motions within and across the protein–protein interface. In addition, continuous cross-interface correlation patterns were observed, suggesting synchronized dynamic communication between SND1 and Ago2 throughout the simulation. Conversely, the SND1MT–Ago2 complex displayed a reduced extent of positively correlated motions together with a greater distribution of negatively correlated regions (blue) (Figure 10B). The cross-interface correlation pattern was less continuous and more fragmented than that observed in the wild-type complex, indicating altered dynamic coupling between the two proteins. These changes were accompanied by an increased prevalence of localized anticorrelated motions, suggesting greater dynamic heterogeneity within the mutant complex.

Overall, the DCCM analysis demonstrated distinct differences in the dynamic correlation patterns between the wild-type and mutant complexes. Compared with the wild-type system, the SND1MT–Ago2 complex exhibited reduced coordinated residue motions and a more heterogeneous distribution of correlated and anticorrelated movements during the simulation, consistent with the altered dynamic behavior observed in the other molecular dynamics analyses presented in this study.

### 2.10. Secondary Structure Analysis

**Figure 11:**
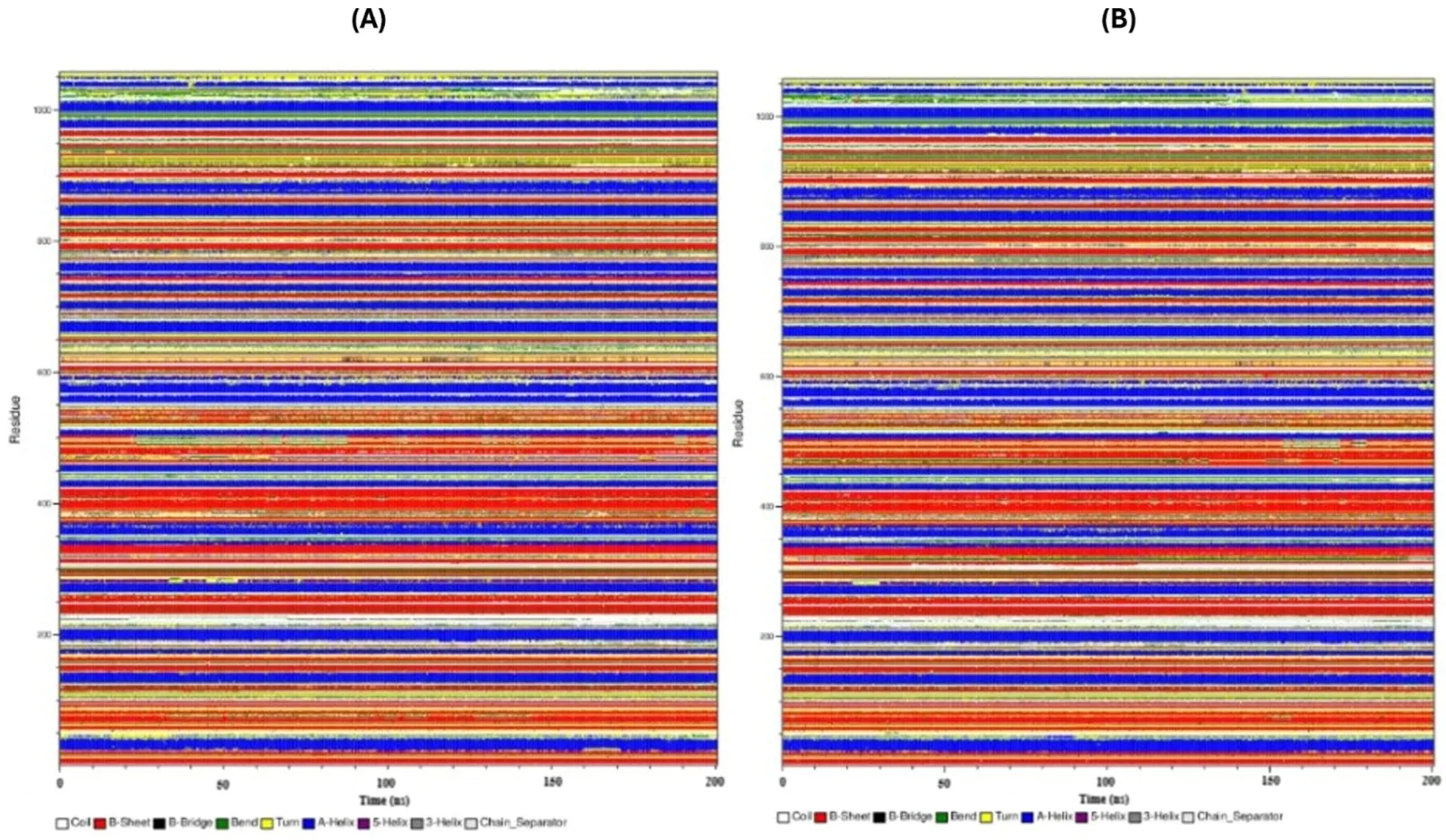
Secondary structure analysis of the SND1–Ago2 complexes during the 200 ns MD simulations. (**A**) Time-dependent secondary structure assignment of the SND1WT–Ago2 complex determined using the Define Secondary Structure of Proteins (DSSP) algorithm. (**B**) Time-dependent secondary structure assignment of the SND1MT–Ago2 complex. Secondary structure elements are color-coded according to the DSSP classification.

The secondary structure evolution of the SND1WT–Ago2 and SND1MT–Ago2 complexes during the 200 ns molecular dynamics simulations was analyzed using the Define Secondary Structure of Proteins (DSSP) algorithm. The analysis monitored the temporal distribution of α-helices, β-sheets, turns, bends, and coil regions throughout the simulation. The SND1WT–Ago2 complex maintained a largely conserved secondary structure during the simulation (Figure 11A). Most α-helical and β-sheet elements remained continuous over time, while only limited transitions among secondary structure elements were observed. The overall secondary structure architecture remained stable throughout the simulation. In contrast, the SND1MT–Ago2 complex exhibited localized changes in secondary structure during the simulation (Figure 11B). Compared with the wild-type complex, the mutant displayed a greater proportion of coil and turn regions together with intermittent interruptions of several α-helical segments. These changes were primarily confined to localized regions, whereas the overall secondary structure framework remained preserved.

Overall, the DSSP analysis indicates that the S781F substitution was associated with localized alterations in secondary structure without causing substantial disruption of the global protein fold. These observations are consistent with the differences in structural dynamics identified by the RMSD, RMSF, hydrogen bond, and principal component analyses.

### 2.11. Molecular Mechanics Poisson–Boltzmann Surface Area Analysis

The binding free energy estimates for SND1WT–Ago2 and SND1MT–Ago2 were predicted using the molecular mechanics Poisson–Boltzmann surface area (MMPBSA) tool, and the results are shown as a violin plot. As shown in Figure S3, a substantial change in binding energy estimate was observed between the two systems. SND1WT–Ago2 showed a higher negative energy estimate (−3002.648 ± 65.896 kJ/mol). On the other hand, a less negative delta G (ΔG) value (−2447.048 ± 63.063 kJ/mol) for SND1MT–Ago2 suggests a probable weaker binding capacity of SND1 and Ago2. This highlights the importance of substitutions in the crucial domain regions. The S781F substitution may possibly tend to influence the local crucial molecular interactions, ultimately leading to changes in binding energy estimates. A possible explanation could be the bulkier, nonpolar nature of phenylalanine. Likewise, Van der Waals, electrostatic, and polar solvation energy values were also calculated (Table 1). A strong and favorable electrostatic interaction (−3486 ± 198.488 kJ/mol) was observed in SND1WT–Ago2, while the interactions were much weaker in SND1MT–Ago2 (−2393 ± 113.493 kJ/mol). In alignment with the electrostatic interaction, polar solvation energy was also computed. For SND1WT–Ago2, the polar solvation energy was 633.641 ± 196.436 kJ/mol, while for SND1MT–Ago2, it was 125.868 ± 127.337 kJ/mol. As observed, the polar solvation resulted in an unfavorable desolvation penalty. Furthermore, per-residue decomposition energy values for the active-site amino acids that could potentially interact with DMA were also analyzed. As shown in Figure S4, the differences between residues in WT and MT are substantial. For instance, the difference in binding energy estimates between the two systems with D742 was around −32.55 kJ/mol, while for D20 and D692, the differences were −20.3 kJ/mol and −56.962 kJ/mol, respectively. N768, which is a key amino acid present in the active site of the Tudor domain, had −0.9088 kJ/mol in WT and 2.398 kJ/mol in the MT system, which once again suggests that the positive value in N768 is unfavorable and thus could not render stabilization for the protein–protein interaction. Overall, the values discussed here should not be interpreted as physical absolute binding free energy values, particularly for a highly charged protein–protein interface, but rather as relative, qualitative estimates of interaction strength within the same modeling framework.

### 2.12. Interaction Analysis Based on Hydrogen Bond Occupancy

To further characterize the intermolecular interactions at the SND1–Ago2 interface, hydrogen bond occupancy analysis was performed over the entire 200 ns MD trajectory (Figure S5). Unlike the time-dependent hydrogen bond analysis presented in Section 2.6, which quantified the instantaneous number of hydrogen bonds formed at each simulation frame, the hydrogen bond occupancy analysis identified the cumulative number of unique residue–residue hydrogen bond interactions sampled throughout the simulation and determined the occupancy of each interaction. Consequently, these two analyses provide complementary information regarding hydrogen bond dynamics and should not be interpreted as measuring the same parameter. The hydrogen bond occupancy analysis identified 35 unique intermolecular hydrogen bond pairs in the SND1WT–Ago2 complex and 76 unique interaction pairs in the SND1MT–Ago2 complex, indicating that the mutant sampled a broader range of residue–residue contacts during the simulation. However, despite the larger number of distinct interaction pairs in the mutant complex, these interactions generally exhibited lower occupancies and were less persistent than those observed in the wild-type complex. This finding is consistent with the time-dependent hydrogen bond analysis (Section 2.6), which showed that the SND1MT–Ago2 complex maintained a lower average number of simultaneous intermolecular hydrogen bonds throughout the simulation.

In the SND1MT–Ago2 complex, the hydrogen bonds with occupancies greater than 25% were Gln669–DMA305 (71.64%), Glu708–Lys328 (27.36%)**,** and Lys709–Val330 (35.82%). In the SND1WT–Ago2 complex, the corresponding high-occupancy interactions were Asp815–Lys107 (55.72%), Val771–Gly304 (34.83%), and Asp692–Lys328 (30.35%), indicating that different residue pairs contributed to the intermolecular hydrogen bond network in the two complexes. Because the Tudor domain of SND1 recognizes methylated arginine residues, hydrogen bond interactions involving DMA in Ago2 were analyzed separately. In the SND1WT–Ago2 complex, six residues (Gln699, Gly704, Thr705, Asp815, Val816, and Gln825) formed hydrogen bonds with DMA. In contrast, eight residues (Glu689, Asp692, Asn768, Arg769, Glu770, Val771, Asp815, and Arg819) interacted with DMA in the SND1MT–Ago2 complex. Although a greater number of residues contacted DMA in the mutant complex, the average hydrogen bond occupancy was substantially higher in the wild-type complex (20.3%) than in the mutant complex (5.5%). Thus, the wild-type complex formed fewer but more persistent DMA-mediated interactions, whereas the mutant complex sampled a larger number of transient contacts with lower occupancies. These results indicate that the various contacts were sampled during the trajectory and should not be considered a direct measure of binding affinity or stability.

Taken together, these findings indicate that the S781F mutation alters the hydrogen bond interaction network by increasing the diversity of intermolecular contacts while reducing their persistence, consistent with the reduced average number of simultaneous hydrogen bonds observed in the time-dependent hydrogen bond analysis. Together, these complementary analyses suggest that the wild-type complex maintains a more stable intermolecular hydrogen bonding network throughout the simulation.

## 3. Discussion

SND1 is a multifunctional RNA-binding protein and an essential RISC component that cooperates with Argonaute proteins in miRNA-mediated silencing. In addition to its role in RNA metabolism, SND1 functions as a transcriptional co-regulator in various cancers [34,35] and has been implicated in multiple cellular processes associated with tumor initiation, progression, invasion, and metastasis [36]. Among the reported genetic alterations in SND1, the S781F substitution is of particular interest because it is located within the Tudor domain, which is responsible for recognizing methylated arginine residues and mediating protein–protein interactions. Replacement of serine with phenylalanine removes the hydroxyl group that can participate in hydrogen bonding and may alter the local physicochemical environment surrounding the interaction interface. Consequently, this substitution has the potential to modify intermolecular interactions and conformational dynamics without necessarily disrupting the overall protein fold. In the present study, computational structural analyses were performed to investigate how the S781F substitution may influence the stability and dynamic behavior of the SND1–Ago2 complex at the atomic level.

The S781F substitution is located within the Tudor domain of SND1, a highly conserved structural module that functions as a reader of methylated arginine and lysine residues and mediates protein–protein interactions involved in RNA metabolism and epigenetic regulation [37]. Tudor domains typically recognize methylated arginine residues through an aromatic binding cage composed of conserved aromatic amino acids that stabilize the methylated ligand via cation–π interactions and complementary hydrogen bonding networks [38,39]. This recognition mechanism is essential for the assembly and regulation of numerous ribonucleoprotein complexes, including those involved in RNA silencing pathways [37]. None of our analyses placed Ser781 itself at the SND1–Ago2 interface. Moreover, Ser781 was not among the aromatic-cage residues that read the dimethylated arginine (Phe740, Tyr746, Tyr763, and Tyr766), and it did not appear on the list of predicted interfacial contact residues obtained from docking (Section 2.1). We therefore do not claim that S781F substitution removed a direct SND1–Ago2 contact. What the data do show is that Ser781 lies within the same extended Tudor module as the binding surface, flanked in sequence by the cage residue Tyr766 on one side and by interfacial contact residues on both sides (Asn768, Arg769, Val771, and Arg776 N-terminal to it; Asp808, Asp815, and Gln822 C-terminal to it), so that a change in side-chain volume and hydrogen bonding capacity at position 781 was structurally positioned to perturb the packing of residues that did contact Ago2. Consistent with a second-shell effect of this kind, the per-residue MM/PBSA decomposition (Figure S4) showed that the contributions of nearby binding-region residues changed between the two complexes. This was most notably observed with Asn768, a key Tudor-domain active-site residue only 13 positions from Ser781, whose contribution shifted from −0.9088 kJ/mol in the wild type to +2.398 kJ/mol in the mutant. Furthermore, Asp742, which was adjacent to the cage residue Phe740, differed by approximately −32.55 kJ/mol between the systems. We therefore emphasize that this is indirect, correlative evidence from a single trajectory per system rather than a demonstration of a causal pathway. Substitution of serine by phenylalanine removes a polar hydroxyl group while introducing a bulkier hydrophobic aromatic side chain, which is expected to modify the local physicochemical environment without disrupting the global Tudor-domain fold. Consistent with this structural context, our molecular dynamics simulations showed that the overall secondary structure of the mutant complex remained largely preserved, whereas localized alterations in residue flexibility, intermolecular hydrogen bonding patterns, and conformational dynamics were observed. These findings suggest that the S781F substitution primarily affects the dynamic organization of the interaction interface rather than causing large-scale structural destabilization. The present computational analyses further indicate that the altered local environment surrounding Ser781 is associated with reduced persistence of intermolecular hydrogen bonds, increased conformational flexibility, and modified correlated residue motions within the SND1–Ago2 complex. Together, these changes are consistent with a less persistent interaction interface and offer a structural rationale for the less favorable MM/PBSA effective binding energy of the mutant complex, which is a relative computational estimate rather than a measured affinity. Nevertheless, because these conclusions are derived from computational modeling, experimental validation using complementary structural and biophysical approaches, such as site-directed mutagenesis, binding affinity measurements, or cryo-electron microscopy, will be required to confirm the molecular consequences of the S781F substitution.

The MD analyses indicate that the S781F substitution primarily alters the conformational dynamics and interaction stability of the SND1–Ago2 complex rather than causing extensive unfolding of either protein. The isolated SND1WT and SND1MT systems showed comparable RMSD, RMSF, radius of gyration, and solvent-accessible surface area values, suggesting that the mutation had only a limited effect on the global structure of unbound SND1. In contrast, the SND1MT–Ago2 complex exhibited a substantially higher backbone RMSD, slightly increased residue-level flexibility and solvent exposure, and a larger radius of gyration than the wild-type complex. The DSSP analysis nevertheless showed that the overall secondary-structure framework remained largely preserved, with the differences principally involving localized increases in coil and turn content and intermittent disruption of selected α-helical regions. Thus, the observed changes are more consistent with localized interface remodeling and altered domain organization than with global protein denaturation. This interpretation is further supported by the PC, free energy landscape, and DCCM analyses. The broader and more dispersed conformational space sampled by the mutant complex, together with its multiple irregular low-energy basins, suggests increased conformational heterogeneity. In parallel, the reduced continuity of positively correlated motions and the increased occurrence of localized anticorrelated motions indicate weakened dynamic coupling between SND1 and Ago2. Previous molecular dynamics studies of disease-associated or function-altering mutations have similarly demonstrated that single-residue substitutions can modify protein flexibility, residue interaction networks, correlated motions, and free energy landscapes without necessarily disrupting the native fold [40,41]. Such effects may arise through changes in local packing, hydrogen-bonding, salt-bridge formation, or solvent interactions and can propagate beyond the mutation site to influence distal regions of a protein or protein–protein interface [41,42]. An integrated elastic-network and protein-structure-network framework for tracing exactly this class of long-range mutational effect has recently been described [43]. Herein, applying it to the SND1–Ago2 system would provide a direct test of the second-shell mechanism proposed above, which the present descriptors can support but not necessarily establish. Taken together, the agreement among RMSD, RMSF, Rg, SASA, PCA, FEL, DCCM, DSSP, and hydrogen bond analyses strengthens the interpretation that the S781F mutation modifies the dynamic stability of the SND1–Ago2 interface. The DCCM and DSSP analyses are based on single trajectories without replicas, and no formal statistical significance testing was performed; these results are therefore interpreted descriptively rather than inferentially. Nevertheless, these descriptors are interrelated measures derived from the same simulated trajectories and should not be regarded as independent experimental confirmation. The findings therefore support a structural hypothesis in which the mutation increases conformational variability and weakens persistent interfacial coupling, but biochemical and biophysical studies will be required to determine whether these predicted changes measurably affect complex formation, dissociation kinetics, or RISC activity.

Although the mutant complex sampled a larger number of distinct intermolecular hydrogen bond pairs during the simulation, these interactions generally exhibited lower occupancies and reduced persistence than those observed in the wild-type complex. This distinction highlights the complementary nature of the two hydrogen bond analyses performed in this study. Whereas the time-dependent analysis quantified the instantaneous number of hydrogen bonds present at each simulation frame, the occupancy analysis identified all unique residue–residue hydrogen bond interactions sampled throughout the trajectory. Therefore, a greater diversity of transient contacts in the mutant complex does not necessarily indicate a more stable interaction network.

As efficient RISC assembly relies on integrated binding of Ago2 with other accessory proteins, perturbations in the SND1–Ago2 interface binding may change the dynamic association of the complex and affect gene silencing. Dysregulation of RISC-associated proteins and RNA-binding proteins has recently gained a huge interest in recognition of its influence on abnormal gene expression and tumor progression [44,45]. Because efficient RISC assembly depends on coordinated interactions between Ago2 and its accessory proteins, perturbations in the modeled SND1–Ago2 interface could, in principle, alter the dynamic organization of the complex and influence the efficiency of miRNA-guided target recognition and gene silencing. However, the present simulations do not directly measure RISC assembly, miRNA activity, or downstream signaling, and these functional implications remain hypothetical. In addition, SND1 has also been noted in several important signaling pathways, which include cell proliferation, invasion, metastasis, and inflammatory responses [46]. Therefore, structural perturbations that may change the SND1 interactions with Ago2 could be crucial. Given the central role of NF-κB in coordinating inflammation-associated tumor progression [44], any mutation-induced disruption of SND1-dependent regulatory networks could potentially influence downstream NF-κB signaling. However, the present study did not directly investigate NF-κB activation, and therefore such mechanistic links should be regarded as biologically plausible hypotheses rather than demonstrated conclusions.

Several methodological limitations should be considered when interpreting these findings. The present study was restricted to the SND1–Ago2 system and therefore did not capture the multicomponent organization of the complete RISC machinery. Moreover, Ser781 was not identified as a direct SND1–Ago2 interfacial residue. Thus, the observed trajectory-dependent differences associated with S781F should be interpreted as potential indirect conformational effects rather than evidence that Ser781 directly determines SND1–Ago2 binding stability. The functional consequences of these changes for RISC activity and cancer-related signaling remain hypothetical and require experimental validation. Each system was represented by a single 200 ns trajectory without independently initialized replicas, and convergence was not established; therefore, the observed differences may partly reflect finite sampling or stochastic divergence. PCA was also performed using separate covariance bases, limiting direct quantitative comparisons of the conformational spaces sampled by the WT and mutant systems. Furthermore, the MM/PBSA analysis was performed without configurational entropy estimation; therefore, the reported energy values should be interpreted as effective binding-energy estimates rather than absolute Gibbs binding free energy values. This limitation, together with the use of single 200 ns trajectories, warrants cautious interpretation of the energetic differences between the two complexes. Future studies should employ multiple independently initialized and longer MD simulations. They also must involve convergence and interface-specific analyses. Lastly, they should employ PCA using a common covariance basis, experimental binding, and functional validation. Further studies should validate the present computational findings using independent replicates and extended MD simulations, alternative protein–protein docking approaches, and experimental SND1–Ago2 binding assays. Comparative simulations of unphosphorylated Ser781, phosphorylated Ser781, and the S781F variant would also help determine whether phosphorylation contributes to SND1–Ago2 recognition. Site-directed mutagenesis and biochemical binding studies could definitively clarify if S781F exerts an indirect second-shell effect on the interaction interface. Finally, functional studies assessing Ago2 recruitment, miRNA-mediated gene silencing, and RISC activity, preferably in the context of additional RISC components, are required to establish the biological significance of the S781F substitution.

## 4. Materials and Methods

### 4.1. Preparation of Protein Structures and Molecular Docking Analysis

The starting coordinates for both proteins were experimentally determined structures deposited in the RCSB Protein Data Bank (PDB). However, no homology models or predicted models were used in this study. For SND1, chain A of PDB entry 3OMC was used, corresponding to the human (*Homo sapiens*) SND1 extended Tudor domain, a 261-residue construct solved at 1.77 Å in complex a symmetrically dimethylated arginine-containing PIWIL1 peptide. For Ago2, chain A of PDB entry 4OLA was used, corresponding to the human Ago2, an 859-residue chain solved at 2.30 Å. Before modeling, crystallographic water molecules and ions were removed from both entries. The co-crystallized product symmetrically dimethylated the PIWIL1 peptide (chains C and D) in 3OMC, whereas the 10-nucleotide RNA (chain B) and crystallization additives were removed from 4OLA. These structural preparation steps were performed using the BIOVIA Discovery Studio 2026. Hydrogen atoms were added, and protonation states were assigned at pH 7.0. Dimethylated arginine305 was introduced into the Ago2 protein using PyMOL Molecular Graphics System, Version 2.5.0 (Schrödinger, LLC, New York, NY, USA) [45]. Similarly, the Ser at position 781 in SND1 was mutated to Phe using the PyMOL software 2.5.0 to generate the S781F mutant (SND1MT) [46]. Protein–protein molecular docking was subsequently performed using the HADDOCK 2.4 Web Server [47,48,49]. The resulting docking poses were ranked according to the HADDOCK scoring function, and representative structures from the highest-ranking clusters were selected for subsequent molecular dynamics (MD) simulations [50].

### 4.2. MD Simulations

MD simulation is a method used to evaluate conformational variations in proteins and their interactions with other ligands or proteins in a simulated environment [51]. A comparative 200 ns MD simulation was carried out for all six systems described in Section 2.2—SND1WT, SND1MT, Ago2, Ago2–DMA, SND1WT–Ago2, and SND1MT–Ago2—using CHARMM-GUI (Chemistry at Harvard Macromolecular Mechanics–Graphical User Interface) [52]. Each system was generated using a solution builder feature and placed in a water box with the TIP3 model [53]. The CHARMM36 force field was used to assess the topologies for each system, and coordinate files were generated [54]. The MD simulations were then performed at a neutral pH and 300 K using the Groningen Machine for Chemical Simulations (GROMACS), Version 2019.4, which was originally developed in the Department of Biophysical Chemistry, University of Groningen, Groningen, The Netherlands, and subsequently maintained by the GROMACS Development Team at the KTH Royal Institute of Technology, Stockholm, and Uppsala University, Uppsala, Sweden [55]. The simple point charge water molecule was then substituted with sodium and chloride ions, hydrogen atoms were added, and protein coordinates were transformed using pdbgmx2 [45]. Further, the electrostatic interaction was determined using the Particle Mesh Ewald method, and the constant numbers of atoms, volume, and temperature, and the constant numbers of atoms, pressure, and temperature of the system were all equilibrated and processed. Accordingly, each system was run for 200 ns [56].

### 4.3. RMSD, RMSF, Rg, SASA and Hydrogen Bond Analysis

The intermolecular H-bond analysis between SND1 and Ago2 in the WT and MT complexes was performed using the gmx H-bond command. Hydrogen bonds were identified using the donor–acceptor distance cutoff of <0.35 nm and hydrogen–donor–acceptor angle cutoff of <30°. By using the CHARMM36 topology files, the donor and acceptor atoms were assigned, and the hydrogen bonds formed between SND1 and Ago2 were considered as intermolecular hydrogen bonds. The hydrogen bond occupancy was then calculated from the analyzed trajectory frames and is shown in Equation (1) as follows:

Occupancy (%) = N_bonded_/N_analyzed_ frames × 100 (1)

where N_bonded_ is the number of analyzed trajectory frames in which that specific H-bond satisfies the defined geometric criteria. N_analyzed_ is the total number of trajectory frames examined for that donor–acceptor pair over the selected analysis period.

### 4.4. DCM and DSSP Analysis

DCCM was calculated based on the MD trajectories using GROMACS tools (gmx covar and gmx anaeig). The periodic boundary conditions were removed, and all frames were fitted to a common reference structure of the corresponding complex by least-squares alignment of C-alpha atoms of their individual systems. The cross-correlation between the fluctuations of residues i and j was determined and shown in Equation (2) as follows:

(2) $$C_{\mathrm{ij}}=\frac{\left({\Delta r}_{i}.{\Delta r}_{j}\right)}{\sqrt{\left({{\Delta r}_{i}}^{2})({{\Delta r}_{j}}^{2}\right)}}$$

where Δr_i_ is the displacement vector of the C-alpha atom of residue I from its average position, and (.) denotes the time average over the trajectory. The resulting matrix elements range from −1 to +1, corresponding to anticorrelated to correlated motion, with values near 0 indicating no correlation. The DCCM was calculated for all the above-mentioned individual systems. But due to the 200 ns trajectories without independent replicas, no statistically significant value could be obtained. Only a difference matrix between the WT and the MT bound to Ago2 could be predicted.

Secondary structure assignments for the above-mentioned complex systems were performed using the DSSP algorithm, which is present in GROMACS (gmx do_dssp). For each frame of the residues, the corresponding residues were classified into alpha-helix, beta-sheets, turn, bend, and coil as per the standard DSSP rules. The time present during the specific frame and its secondary structure state for each system was predicted. As in other experiments, only single 200 ns trajectories were present and were used for the analysis.

### 4.5. Principal Component and Free Energy Landscape Analysis

PCA was performed using the MD simulation trajectories and GROMACS tools (gmx covar and gmx anaeig). Firstly, the periodic boundary conditions were removed, and all the frames were fitted to a common starting frame of the corresponding complex by least-squares alignment of C-alpha atoms. In this study, PCA was performed separately for the SND1WT and SND1-S781F trajectories using their own matrix and eigenvalues. A similar process was carried out for the Ago2 WT and MT complexes. Due to this, the PC1 and PC2 coordinates are represented only in a qualitative manner and not quantitatively, as each trajectory corresponds to a different eigenvector. Thus, the PC1 and PC2 projections chosen here were then further used for the qualitative illustration of the free energy landscape (FEL) plots by F(PC1, PC2) = −k_B_Tln P(PC1, PC2) + C, where P stands for normalized two-dimensional probability density, T = 300 K, and C is a constant indicating that minimum free energy is zero. Because the FELs were derived from single 200 ns trajectories with separate covariance bases and finite sampling, they are interpreted as qualitative conformational-density maps rather than quantitative thermodynamic free energy surfaces.

### 4.6. MM/PBSA Analysis

The trajectory analysis was performed using specific GROMACS utilities such as gmx_rmsd, gmx_rmsf, gmx_gyrate, and gmx_hbond [57]. The MMPBSA approach was employed to understand the binding free energy over simulation time [58]. A GROMACS utility, g_mmpbsa, was employed to estimate the binding free energy [58]. To obtain an accurate result, we computed the last 50 ns with a dt of 500 frames [59]. The polar solvation term was obtained by solving the Poisson–Boltzmann equation with a solute dielectric constant of 4, a solvent dielectric constant of 80, an ionic strength of 0.15 M, and a grid spacing of 0.5 nm. Atomic radii were assigned according to the CHARMM parameter set.

This program uses a Python script, MmPbSaStat.py, to predict the average energy for each system [37]. The ΔG was further calculated as per the following equation:

ΔG_binding_ = G_complex_ − (G_protein_ + G_ligand_) (3)

G_complex_ is the free energy of the SND1–Ago2 complex, and G_protein_ and G_ligand_ represent the free energy values of the two separated binding partners. In the present protein–protein application, these correspond to free SND1 and free Ago2, respectively [38]. The estimation of free energy in both bound and unbound forms was determined by employing the following equation:

G_x_ = (EMM) − TS + (G_solv_) (4)

where G_x_ corresponds to either the SND1–Ago2 complex or one of the free binding partners, molecular mechanics energy (EMM) is the average molecular mechanics energy, TS is the entropic contribution, and G_solv_ is the solvation free energy of the corresponding species [32]. It should be noted that the T_S_ term is shown here for completeness of the underlying formalism only because configurational entropy was not calculated in this work, and the reported values are accordingly MM/PBSA effective binding energy values rather than complete Gibbs binding free energy values [60].

The EMM value was calculated by considering the electrostatic and van der Waals interactions between SND1 and Ago2 within the complex, as shown in Equation (5) [32]. G_solv_ denotes the linear Poisson–Boltzmann equation for individual states (G_polar_), and the solvent-accessible surface area was estimated using non-hydrophobic interactions.

EMM = E_bonded_ + E_non–bonded_ (5)

G_solv_ = G_nonpolar_ + G_polar_ (6)

The g_mmpbsa tool was employed to predict the binding energy values from the MMPBSA run. Following this, the “MmPbsaDecomp.py” script was used to determine the overall binding energy contributed by each targeted individual amino acid [32]. No configurational entropy term was calculated for any system. Moreover, normal-mode and interaction-entropy analyses were also not performed. Consequently, the reported MM/PBSA values represent effective binding energy values rather than complete Gibbs binding free energy values. Because the same single-trajectory MM/PBSA protocol and level of approximation were applied consistently to both complexes, the calculated energy values were used only for relative comparisons between the SND1WT–Ago2 and SND1MT–Ago2 systems.

## 5. Conclusions

The present study employed an integrated computational approach combining protein–protein docking, molecular dynamics simulations, and free energy analyses to investigate the structural consequences of the SND1 S781F substitution on the interaction between SND1 and Ago2. Compared with the wild-type complex, the SND1MT–Ago2 complex showed changes in interfacial hydrogen bond patterns, residue fluctuations, correlated motions, and sampled conformational space, whereas the overall protein fold remained largely preserved. These observations support a hypothesis of localized interface remodeling and varied conformational dynamics. The findings of this study, however, do not directly demonstrate any reduced equilibrium affinity, altered binding kinetics, improper RISC assembly, or changes in miRNA-mediated silencing. Furthermore, they do not indicate evidence of Nf-kB activation or of being tumor-driven. The results simply give an assumption and should be considered hypothesis-driven. Future studies should require validation through experimental methods such as co-immunoprecipitation, SPR-based quantitative binding assays or ITC, and in vitro-based cellular functional assays.

## Figures and Tables

**Table 1:** MM/PBSA energy components for the SND1WT–Ago2 and SND1MT–Ago2 complexes. Values are presented as mean ± SD (kJ/mol).

System	Binding Energy (kJ/mol)	Electrostatic Energy (kJ/mol)	Polar Solvation Energy (kJ/mol)
SND1WT–Ago2	−3002.648 ± 65.896	−3486 ± 198.488	633.641 ± 196.436
SND1MT–Ago2	−2447.048 ± 63.063	−2393 ± 113.493	125.868 ± 127.337

## Data Availability

The original contributions presented in this study are included in the article/Supplementary Material. Further inquiries can be directed to the corresponding authors.

## Supplementary Materials

The following supporting information can be downloaded at: https://www.mdpi.com/article/10.3390/ijms27198747/s1.

## Abbreviations

The following abbreviations are used in this manuscript:

Ago2	Argonaute-2
AKT	Protein kinase B
BSA	Buried Surface Area
CHARMM-GUI	Chemistry at Harvard Macromolecular Mechanics–Graphical User Interface
DCCM	Dynamic cross-correlation matrix
DSSP	Define Secondary Structure of Proteins
EMM	Molecular mechanics energy
FEL	Free energy landscape
FOXO3a	Forkhead box O3a
GROMACS	GROningen MAchine for Chemical Simulations
HADDOCK	High Ambiguity Driven Biomolecular DOCKing
MD	Molecular dynamics
miRNA	Micro-ribonucleic acid
DMA	Dimethylated arginine
MMPBSA	Molecular mechanics Poisson-Boltzmann surface area
MT	Mutant
NF-κB	Nuclear factor kappa B
PC	Principal component
PDB	Protein Data Bank
PI3K	Phosphatidylinositol 3-kinase
PTEN	Phosphatase and tensin homolog
Rg	Radius of gyration
RISC	RNA-induced silencing complex
RMSD	Root mean square deviation
RMSF	Root mean square fluctuation
SASA	Solvent accessible surface area
SND1	Staphylococcal nuclease domain-containing protein 1
WT	Wild type
ΔG	Gibbs free energy change
