# Supplementary material for: Computational Investigation of S781F Mutation-Induced Remodeling of the Staphylococcal Nuclease Domain-Containing Protein 1–Argonaute 2 Interface
Source: Int J Mol Sci. 2026 Sep 30;27(19):8747. doi: 10.3390/ijms27198747 (PMC13664806; doi:10.3390/ijms27198747)
Supplement: Supplementary file 1 [file ijms-27-08747-s001.zip › ijms-4507709-supplementary.pdf]

Supplementary Information

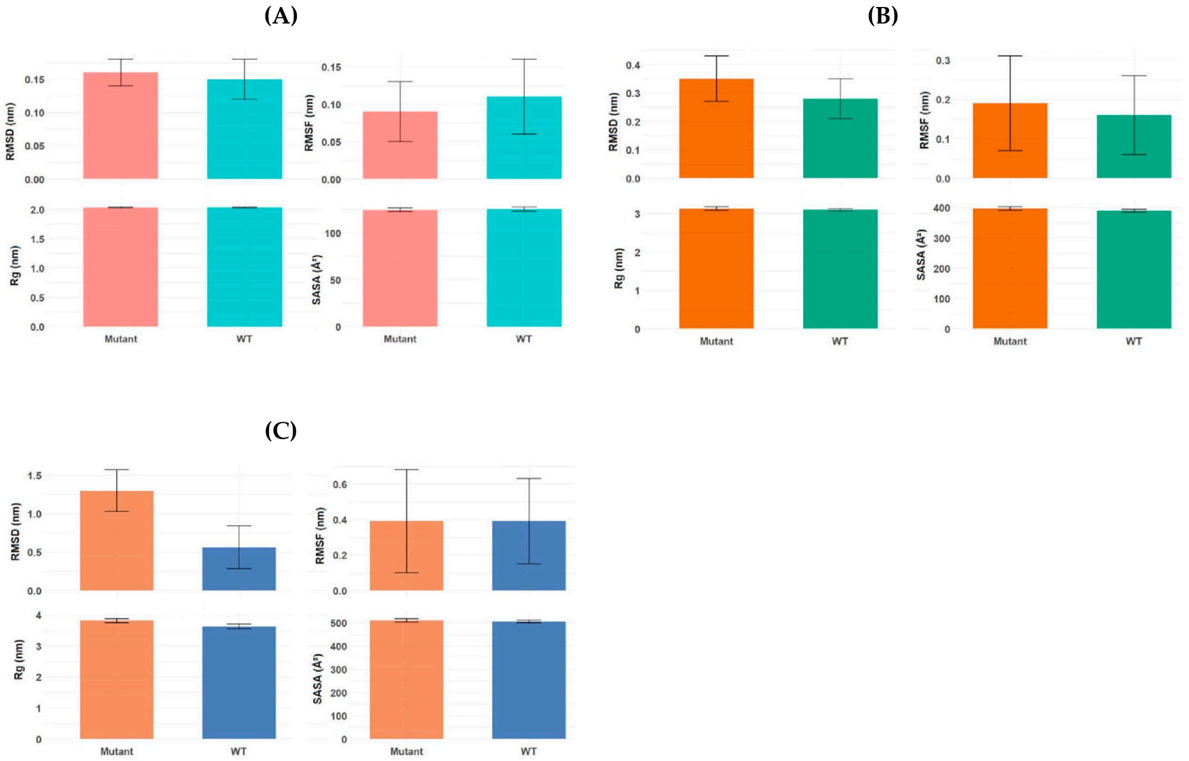

Figure S1. Structural stability analysis of the wild-type staphylococcal nuclease domain-containing protein 1 (SND1WT) and S781F mutant staphylococcal nuclease domain-containing protein 1 (SND1MT)-Argonaute 2 (Ago2) complexes during the molecular dynamics (MD) simulations. **(A)** Comparison of average backbone root mean square deviation (RMSD). **(B)** Comparison of radius of gyration (Rg). **(C)** Comparison of residue root mean square fluctuation (RMSF). Error bars represent the standard deviation calculated from the simulation trajectories.

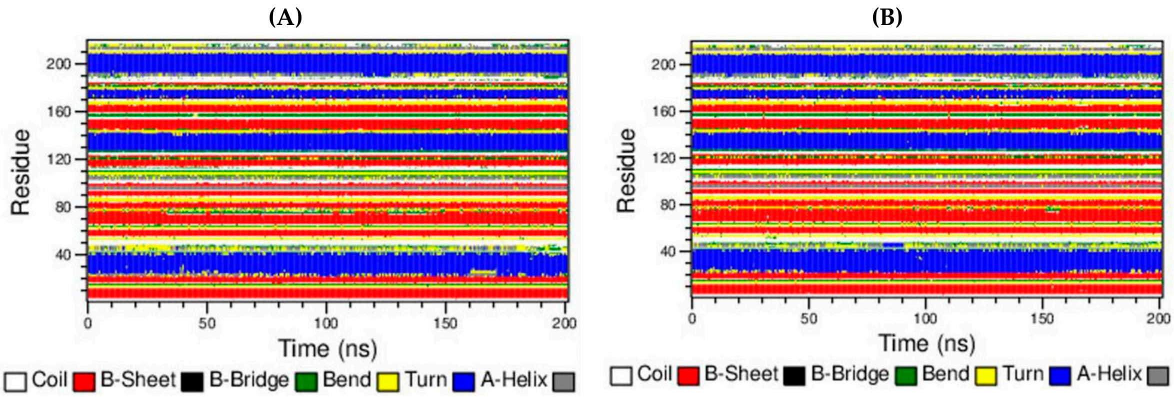

Figure S2. Secondary structure analysis of SND1WT and SND1MT during the MD simulations. **(A)** Time-dependent secondary structure assignment of SND1WT determined using define secondary structure of protein (DSSP). **(B)** Time-dependent secondary structure assignment of SND1MT determined using DSSP. Secondary structure elements are color-coded according to the DSSP classification.

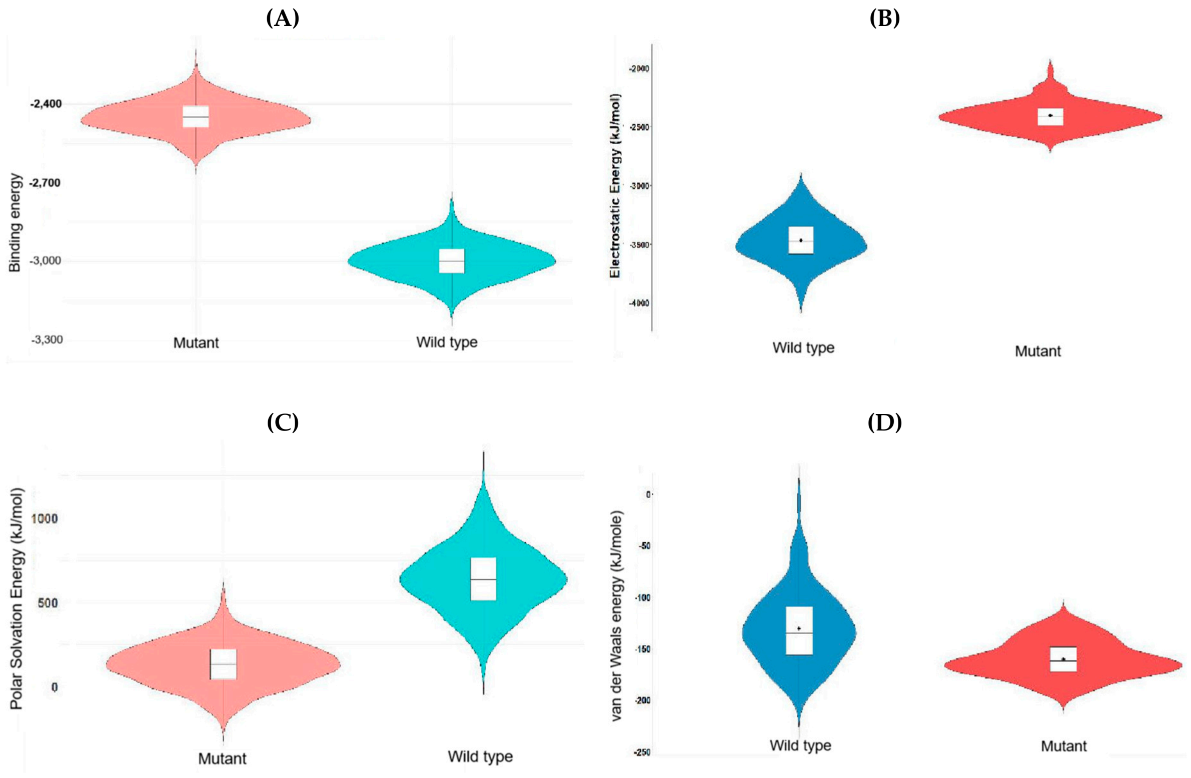

Figure S3: Molecular Mechanics/Poisson-Boltzmann surface area (MM/PBSA) effective interaction-energy analysis of the SND1WT-Ago2 and SND1WT–Ago2 complexes. **(A)** Total MM/PBSA effective-energy estimates. **(B)** Electrostatic energy contributions. **(C)** Polar solvation contribution; spositive values represent unfavorable desolvations. **(D)** van der Waals energy contributions. Values are derived from single MD trajectories and should not be interpreted as absolute Gibbs binding free energies.

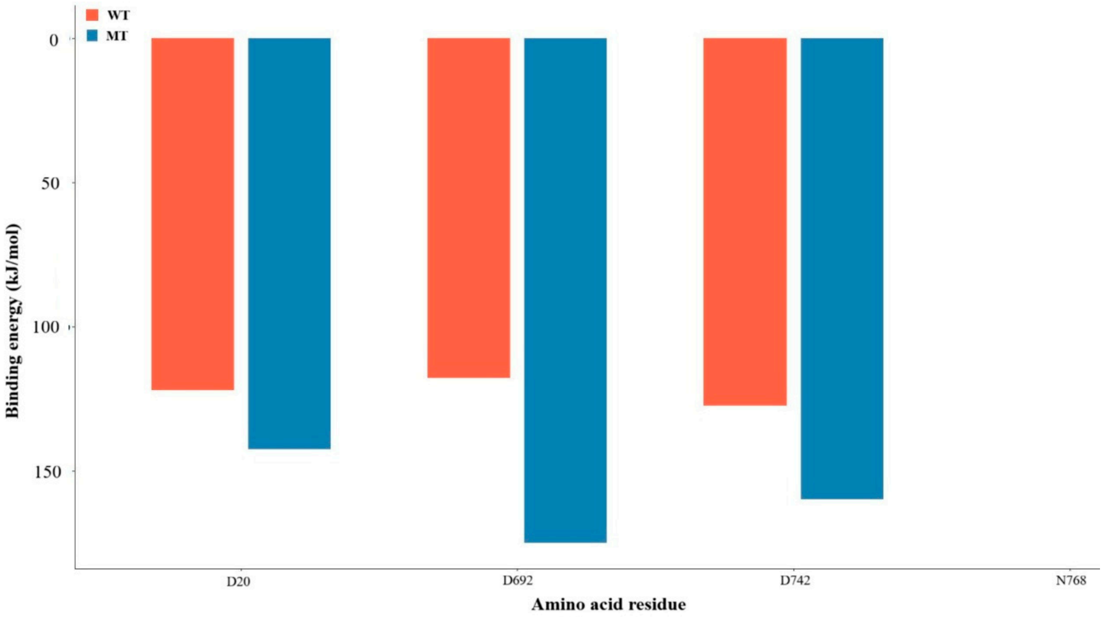

Figure S4. Per-residue MM/PBSA energy decomposition for the SND1WT–Ago2 and SND1MT-Ago2 complexes. Residue-wise values are interpretative partitions of the calculated effective interaction energy and are not rigorous residual-specific binding free energy.

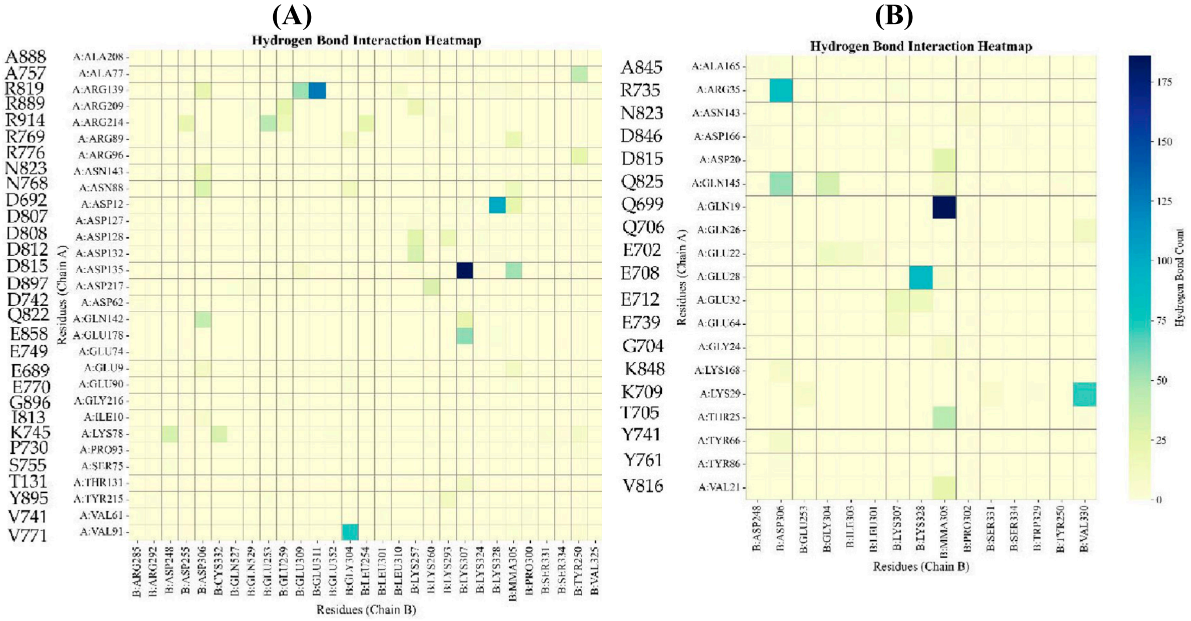

Figure S5: Hydrogen-bond occupancy at the SND1–Ago2 interface during the 200 ns MD simulations. **(A)** SND1WT–Ago2 complex. **(B)** SND1MT–Ago2 complex. Color intensity represents the percentage of analysed frames in which each donor-acceptor pairs satisfied the hydrogen-bond geometric criteria.

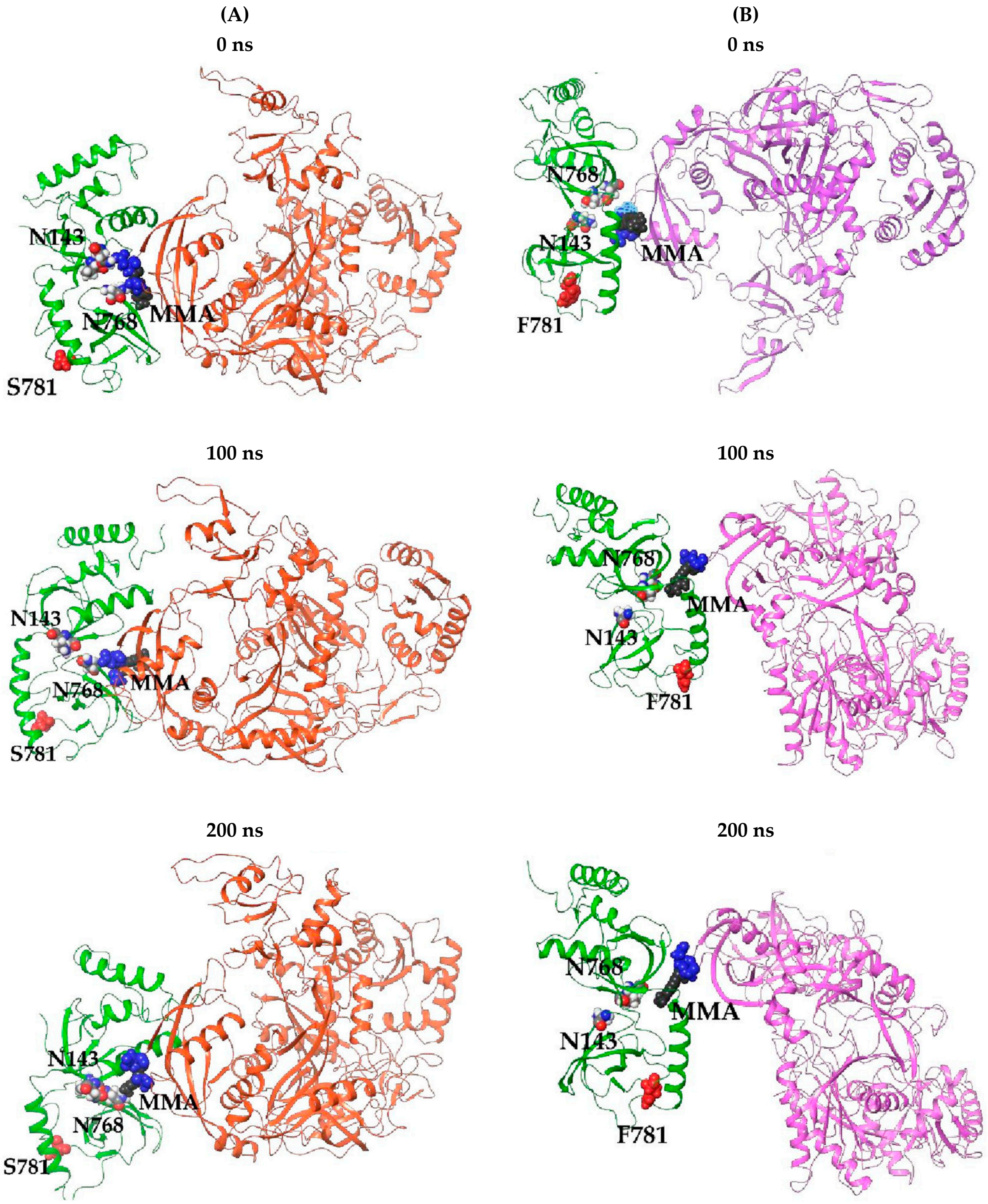

Figure S6. Representative conformations of the SND1–Ago2 complexes during the MD simulations **(A)** SND1WT–Ago2 complex snapshots at 0, 100, and 200 ns. **(B)** SND1MT–Ago2 complex snapshots at 0, 100, and 200 ns.
